# The Mars Organic Molecule Analyzer (MOMA) Instrument: Characterization of Organic Material in Martian Sediments

**DOI:** 10.1089/ast.2016.1551

**Published:** 2017-07-01

**Authors:** Fred Goesmann, William B. Brinckerhoff, François Raulin, Walter Goetz, Ryan M. Danell, Stephanie A. Getty, Sandra Siljeström, Helge Mißbach, Harald Steininger, Ricardo D. Arevalo, Arnaud Buch, Caroline Freissinet, Andrej Grubisic, Uwe J. Meierhenrich, Veronica T. Pinnick, Fabien Stalport, Cyril Szopa, Jorge L. Vago, Robert Lindner, Mitchell D. Schulte, John Robert Brucato, Daniel P. Glavin, Noel Grand, Xiang Li, Friso H. W. van Amerom

**Affiliations:** ^1^ Max-Planck-Institut für Sonnensystemforschung, Göttingen, Germany.; ^2^ NASA GSFC, Greenbelt, Maryland, USA.; ^3^ LISA, U. Paris-Est, Créteil, U. Paris Diderot, Paris, CNRS, France.; ^4^ Danell Consulting, Winterville, North Carolina, USA.; ^5^ RISE Research Institutes of Sweden, Bioscience and Materials/Chemistry and Materials, Stockholm, Sweden.; ^6^ LPGM, CentraleParis, Chatenay-Malabry, France.; ^7^ LATMOS/IPSL, Guyancourt, France.; ^8^ University of Maryland, College Park, Maryland, USA.; ^9^ Université de Nice-Sophia Antipolis, Nice cedex, France.; ^10^ Institut Universitaire de France, Paris, France.; ^11^ ESA, Noordwijk, The Netherlands.; ^12^ NASA Headquarters, Washington, DC, USA.; ^13^ INAF—Astrophysical Observatory of Arcetri, Firenze, Italy.; ^14^ University of Maryland, Baltimore County, Baltimore, Maryland, USA.; ^15^ Mini-Mass Consulting, Hyattsville, Maryland, USA.; ^16^ University of Bremen, Bremen, Germany.

## Abstract

The Mars Organic Molecule Analyzer (MOMA) instrument onboard the ESA/Roscosmos ExoMars rover (to launch in July, 2020) will analyze volatile and refractory organic compounds in martian surface and subsurface sediments. In this study, we describe the design, current status of development, and analytical capabilities of the instrument. Data acquired on preliminary MOMA flight-like hardware and experimental setups are also presented, illustrating their contribution to the overall science return of the mission. Key Words: Mars—Mass spectrometry—Life detection—Planetary instrumentation. Astrobiology 17, 655–685.

## Table of Contents

1. Introduction: The Mars Organic Molecule Analyzer (MOMA) Investigation

2. Science Goals

3. Objectives and Requirements

4. Instrument Top-Level Description

5. Instrument Development

5.1. Team organization

5.2. Instrument integration flow

5.3. Instrument verification

5.4. Achieved milestones and deliveries

6. Instrument Subsystems

6.1. Oven and tapping station subsystems

6.1.1. Derivatization

6.1.1.1. MTBSTFA

6.1.1.2. DMF-DMA

6.1.2. Thermochemolysis

6.1.3. Wet chemistry: storage and release of chemical agents

6.2. Gas chromatograph subsystem

6.2.1. Design requirements

6.2.2. Design overview

6.2.2.1. General architecture

6.2.2.2. Injection system

6.2.2.3. Separation of volatiles

6.2.2.4. Detection of volatiles

6.2.2.5. Gas handling system

6.2.2.6. GCMS coupling campaigns

6.3. Laser subsystem

6.3.1. Design requirements

6.3.2. Laser architecture

6.3.3. Verification data

6.4. Mass spectrometer subsystem

6.4.1. Design requirements

6.4.2. Analyzer architecture

6.4.2.1. Linear Ion Trap assembly

6.4.2.2. Detection channels

6.4.2.3. Electron Ionization Source

6.4.2.4. Fast-actuating aperture valve

6.4.2.5. Micropirani pressure sensor

6.4.2.6. Wide-Range Pump

6.4.3. Temperature control

6.4.4. Driving electronics

7. Operation of MOMA on the Surface of Mars

7.1. Expected measurement scenarios

7.2. Sequenced operational modes

7.3. Synergy with other instruments

7.3.1. Data interpretation

7.4. LDMS operations on Mars and test experiments on LDMS prototype system

7.5. Test experiments on GCMS prototype system: a case study

7.5.1. Sample and setup

7.5.2. Methods

7.5.2.1. Stepwise pyrolysis

7.5.2.2. Derivatization/thermochemolysis

7.5.2.3. GCMS parameters

7.5.3. Results and discussion

7.5.3.1. Pyrolysis

7.5.3.2. Derivatization and thermochemolysis

8. Discussion

9. Summary

Acknowledgments

Author Disclosure Statement

References

Abbreviations Used

## 1. Introduction: The Mars Organic Molecule Analyzer (MOMA) Investigation

The Mars Organic Molecule Analyzer (MOMA) investigation directly addresses the ExoMars scientific objective (Vago *et al.*, 2016) to search for signs of past or present life on Mars. It achieves this by analyzing a wide range of organic compounds that may be found in drill samples acquired up to 2 m below the martian surface. MOMA must first volatilize organic compounds so that they can be detected by a mass spectrometer (MS). Volatilization of organic material is achieved by either one of its two operational modes: (1) heating of the sample to induce evaporation and/or thermochemical decomposition (pyrolysis) and liberate species into the gas phase, possibly also being preceded by a chemical derivatization step to aid in this gas-phase transition, and (2) direct interrogation of the sample by intense ultraviolet (UV) laser pulses inducing prompt desorption into the gas phase. In the case of operational mode (1), the organic compounds will be separated by gas chromatographic columns before they reach the MS, while in the case of operational mode (2), the laser-desorbed species are sent directly to the MS without further separation. Either mode enables direct detection of indigenous martian organic molecules (in some cases, as unfragmented parent ions) and will thus be of high diagnostic and scientific value. MOMA can also detect some thermally released inorganic molecules (*e.g.*, SO_2_) or laser-desorbed fragments of inorganic minerals (*e.g.*, iron oxide or silicate fragments). By characterizing the types, distributions, and molecular structures of detected organics, MOMA can provide powerful insights into the origin and processing of potential molecular biosignatures.

While the existence of a singular, unambiguous martian biosignature may be endlessly debated, a common pragmatic position—that taken by ExoMars—is that a martian biosphere would be reflected to some degree in the distribution and structures of organic compounds associated with past or present organisms (Summons *et al.*, 2008; Westall *et al.*, 2015). For example, certain lipids may be produced by the chemical and thermal degradation of biomolecules forming cell membranes and can be preserved over geological timescales (Brocks and Summons, 2003). The aliphatic groups of these and other organics may also exhibit biogenic features, such as a strong even-over-odd bias in fatty acid carbon chain lengths, which are not present in abiogenic organic material (*e.g.*, McCollom, 1999; Peters *et al.*, 2005). If biogenic, such compounds may be found at high concentrations only over a discrete range of molecular weights (such as the C_14_–C_20_ fatty acids). This is in contrast to that observed in carbonaceous meteorites where these compounds are detected over a broader range of molecular weights (typically hundreds of atomic mass units) and with decreasing abundance as molecular weight increases (Sephton, 2002; Summons *et al.*, 2008). Molecular chirality (handedness) is another important property of some organic compounds (*e.g.*, amino acids and sugars) that can be used to discriminate between biological and nonbiological sources if there is shown to be a strong enantiomeric excess (ee) (Meierhenrich, 2008). Particular isomers of high-molecular-weight organics (10^2^–10^3^ u), specifically those with identifiable biochemical functionality such as terpenoids and peptides, can be potential biosignatures when identified in concert with other supporting evidence.

MOMA has been designed to enable an investigation of such molecular signs of life—extinct or extant—through a combination of *detection and characterization* of key complex organic compounds and *contextual measurements* of organic and inorganic inventories. Understanding these broader inventories, by using combined data from MOMA and other ExoMars instruments, can provide a more complete understanding of active processes, such as organic delivery, degradation, and cycling, as well as the preservation potential of the local environment.

Several regions on Mars have been advocated as appropriate exploration areas for the ExoMars rover mission. These regions either show fine-grained sedimentary deposits (*e.g.*, clay minerals) that may adsorb and trap organic material due to their high specific surface area or the deposits have been rapidly buried (*e.g.*, by lava) and later exposed by slow and still ongoing Amazonian erosion. The latter process would have shielded organic compounds against destructive cosmic particle radiation (Goetz *et al.*, 2016b, and references therein). As of April 2017, two candidate landing sites are being considered: Oxia Planum and Mawrth Vallis (Vago *et al.*, 2017). Both areas are located in western Arabia Terra (15°–25°N, 15°–25°W, less than 500 km from each other), off the dusty terrain, and at altitudes (−3 to −2 km) compatible with EDL (Entry, Descent, and Landing) constraints (Vago *et al.*, 2017). Both locations lie in a clay rich late-Noachian/early-Hesperian region that was covered by an Amazonian lava flow (c. 2.6 Ga). Since then, denudation and scarp retreat have been ongoing, providing access to old sediments that may have preserved pristine Noachian organic compounds (Carter *et al.*, 2016; Quantin *et al.*, 2016).

## 2. Science Goals

The overarching goal of MOMA is to seek the signs of past or present life on Mars through analysis of the molecular, primarily organic, composition of acquired samples on the ExoMars rover. This overarching goal is divided into three subgoals, here updated for clarity from the work by Debus *et al.* (2012), as follows:

*G1. Search for molecular biosignatures*: A molecular biosignature is a pattern or distribution of molecules or molecular structures that results from biological processes, past or present. Molecular, primarily organic, biosignatures may represent or derive from parts of the organisms themselves, such as cell wall lipids, or reflect the products of life processes, such as biogenic methane. On Earth, such biosignatures are ubiquitous and recognizable. On Mars, molecular structures, let alone those associated with viable organisms, face what appear to be severe conditions likely rendering them more obscure. Degradational processes, such as radiolysis and oxidation that dominate near the surface, may leave only ambiguous remnants of a potential biosignature. However, under favorable taphonomic conditions (*e.g.*, rapid burial), certain biosignatures such as isomeric and abundance biases might be preserved on Mars, even over geologic timescales. As such, the search for evidence of molecular biosignatures by MOMA must be broad, able to detect the widest range of molecular structures, and must be interpreted in the context of both preservation potential and an expected background of abiotic chemistry.

The focus of ExoMars is on signs of ancient life. Under appropriate geological conditions, such as rapid sedimentary burial and limited oxidation potential, martian molecular biosignatures could be detectable through their diagenetic products, distinct from an abiotic (*e.g.*, exogenous) organic background. Certain classes of molecular fossils such as acetogenic and isoprenoidal lipids, carotenoids (such as β-carotene), and key biomarker species, such as pristane, phytane, and hopanes, have been studied for their specificity to terrestrial microorganisms and their ability to be preserved over long timescales (Summons, 2014). Both modes of MOMA, separately and in concert, may contribute critical data toward their identification. The Laser Desorption Mass Spectrometry (LDMS) mode may provide initial indication of higher-molecular-weight (hundreds of atomic mass units) nonvolatile organics, consistent with such compounds, which can be structurally analyzed through subsequent tandem mass spectrometry as well as derivatization- or thermochemolysis-based Gas Chromatography–Mass Spectrometry (GCMS). Moreover, when coupled with the mission's ability to analyze depth profiles, the molecular weight distribution of a wide variety of organics, from amino and carboxylic acids, to polycyclic aromatic hydrocarbons (PAHs) and their alkylated homologs, to larger moieties such as porphyrin-based species and macromolecular carbon, covered by MOMA's broad-based detection approach, may help resolve the provenance of any detected organics.

One of the more important signatures in terrestrial biochemistry involves the use of only one enantiomer of amino acids as monomers for peptide synthesis, referred to as homochirality. A significant left or right imbalance or ee (defined as ([L]−[R])*100%/([L]+[R]) where [L] and [R] refer to the abundance levels of, respectively, left and right enantiomers), detected with the appropriate chemical derivatization tag and a chiral-selective GC column, represents a *potential* molecular biosignature. Abiotic processes such as UV polarized radiation and surface catalysis on specific crystals are generally only known to produce tiny ee of any sign and up to 1% (Modica *et al.*, 2014). However, significant ee (up to +60%) has been observed for certain amino acids in carbonaceous chondrites, implying the existence of prebiotic mechanisms to amplify tiny initial ee (Glavin and Dworkin, 2009; Glavin *et al.*, 2012, and references therein; Tarasevych *et al.*, 2015); any positive ee detection by MOMA would thus require additional correlative evidence from a suite of measurements. In particular, a significant (say, greater than 20%) L-excess in a suite of martian amino acids, although potentially a compelling observation, would require further corroborating chemical evidence. Similarly, a very significant D-excess would be a remarkable finding as this could indicate a biochemistry entirely independent from that of Earth.

Molecular biosignatures caused by extant or very recent (dormant or extinct) biology on Mars would be more pronounced and thus more easily detectable than those caused by ancient biology. However, the ExoMars rover mission (as opposed to the Viking landers, Goetz *et al.*, 2016b, and references therein) is not designed to distinguish between signs of current and past life. Absent a parallel direct search for metabolism or cells and organisms, it will be difficult to distinguish extant versus extinct potential molecular biosignatures in isolation without a full understanding of the taphonomic conditions of any particular structure or pattern, which is not well constrained for the martian near-subsurface.

Nevertheless, due to the predicted degradation rates for certain signatures, based on terrestrial biology and laboratory observations, detection of certain classes of complex organics, in the context of other measurements, could provide some evidence of potential extant or recent biology. Assuming martian life would be carbon-based and cellular as on Earth, one would expect common or analogous biomolecular building blocks such as chains of amino acids (peptides and proteins or their analogs) and oligomers of nucleobases (RNA, DNA, or their analogs such as PNA—peptide nucleic acid [Nelson *et al.*, 2000; Zimmer, 2009]). Such larger, more fragile polymeric biomolecules are less likely to survive environmental processes and could indicate recent biosynthesis. Similarly, phospholipids would be prevalent in the cell membranes of any viable community of bacteria or archaea, and the internal lipid structure could be used to distinguish among these (*e.g.*, isoprenoid side chains in archaea). Individual compound classes targeted for detection would thus include fatty acids, sterols, and hopanoids. Strong evidence of ee in amino acids within such a population may also be an indicator of current or recent biological activity due to the tendency for racemization over geologic time (Bada and McDonald, 1995; Bada *et al.*, 1999).

*G2. Search for evidence of active processes (geochemical, biologic, or exogenous):* The astrobiological interpretation of the martian organic inventory, as it varies over spatial and temporal dimensions, requires a broad understanding of its carbon and other chemical processes and cycles. Such an understanding is only now beginning to emerge with a synthesis of results from multiple missions, including MER, MEX, MSL (Curiosity rover), and MAVEN (see the Abbreviations Used section for expansion of mission acronyms). The ExoMars rover, with MOMA, has the opportunity to achieve a new view of Mars, with its combination of depth sampling, likely late Noachian terrain, and MOMA's sensitivity to a variety of molecules over a range of volatilities and molecular weights. Detection of compounds with relatively weak bonds, such as C-O-C or C-N-C, in the harsh near-surface environment implies replenishment over a given depth-dependent timescale or high erosion rates/recent exposure. Abundance levels of such species significantly exceeding those expected from exogenous infall (Steininger *et al.*, 2012, and references therein; in particular Flynn and McKay, 1990), correlated with any higher-molecular-weight organics containing such bonds, may comprise a potential biomarker. Lighter, volatile, and semivolatile compounds (with enthalpies of vaporization below ∼50 kJ/mol), including *n*-alkanes, some amino acids, benzene, and even naphthalene and benzoic acid, detectable by the GCMS mode, may provide insights into active processes (*e.g.*, recent exposure due to sublimation of surface ice or eolian denudation) that broadly determine the organic inventory. Additionally, the surface of Mars should have accumulated significant quantities of larger more refractory organics based on the abundance levels found in interplanetary dust particles and carbonaceous meteorites. MOMA's characterization of this fraction, primarily by LDMS, may determine not only the abundance of this potential background for trace biomarker detection but also the degree of processing of this matter by radiation (Moores and Schuerger, 2012; Pavlov *et al.*, 2012; Poch *et al.*, 2014, 2015) and oxidation (Benner *et al.*, 2000) as a function of depth. Some refractory material can also be volatized by thermochemolysis or derivatization and thereby analyzed with GCMS.

*G3. Understand the geologic context:* MOMA is primarily focused on organic compounds. Important ExoMars objectives related to determining geological and geochemical context through inorganic composition are well handled by other highly capable instruments. Nevertheless, MOMA does possess some capabilities for inorganic elemental and mineralogical analysis that complement those instruments. The presence or absence of certain elements (including S, Ca, and some transition metals) and of general mineral classes (*e.g.*, carbonates, sulfates, sulfides, iron oxides) provides important contextual information about the processing and chemical complexity of the material, as well as correlation with any detection of organic compounds in the same sample. In the LDMS mode, diagnostic laser-desorbed metal ions, metal clusters, or metal oxide molecules (with characteristic isotope patterns) are produced during the standard analysis. While not part of the baseline investigation, MOMA does additionally have the capability to perform both evolved gas analysis (EGA), by monitoring the release of small molecules (*e.g.*, SO_2_, OCS, CS_2_) as a function of temperature, and differential thermal analysis (DTA; Goetz *et al.*, 2011; Archer *et al.*, 2013) by comparing EGA temperature ramp data from two successive runs of a common sample at exactly the same oven power profile. EGA has been conducted very successfully by the SAM instrument (sample analysis at Mars) onboard the Curiosity rover (McAdam *et al.*, 2014, 2016; Sutter *et al.*, 2016). Differential scanning calorimetry (DSC) experiments were successfully conducted by TEGA (thermal and evolved gas analyzer) onboard the Phoenix Mars lander (Boynton *et al.*, 2009; Sutter *et al.*, 2012). The latter technique (DSC) consists of measuring the differential energy needed to increase the sample temperature by a small amount (denoted as Δ*E*/Δ*T*) at any time while the sample is heated. All thermal analysis techniques described so far (EGA, DTA, DSC) are not part of MOMA instrument requirements (Section 3) and would therefore be performed on a best effort basis based on the rover's science operation team decision process. Overall, MOMA's constraints on the chemical and mineralogical composition of samples must be combined with data from the RLS (Raman Laser Spectrometer) and MicrOmega (Micro Observatoire pour la Mineralogie, l'Eau, les Glaces et l'Activité, *i.e.*, NIR imaging spectrometer) instruments. The fine focus of the MOMA laser enables qualitative association of any organic compounds with the mineral matrix.

## 3. Objectives and Requirements

To achieve these goals, MOMA is tasked by ExoMars with four analytical objectives as follows:

O1. Detect and characterize organic molecular species in solid samples with high (ppb) sensitivity.
O2. Analyze patterns of, and interrelationships among, a variety of organic molecules over a wide range of molecular weights and volatilities.
O3. Analyze for the presence and degree of ee in detected chiral organics.
O4. Characterize the inorganic geochemical context of the organic analyses.

These objectives translate into a set of performance requirements (Debus *et al.*, 2012) that have dictated MOMA design and development:

R1. MOMA shall be able to detect organic molecules in the collected samples at concentrations as low as 10 parts-per-billion by weight (ppbw) or ng/g.

R2. MOMA shall be able to identify organics with chain-based or ring-based structures, which could indicate potential biosignatures, at concentrations as low as 10 ppmw.

R3. MOMA shall be able to characterize molecular weight distribution patterns in organic molecules (*e.g.*, odd vs. even chained hydrocarbons, heteroatom, or methyl substitution in aromatics) over mass-to-charge (*m/z*) ratio as high as 1000 u at concentrations as low as 10 ppmw.

R4. MOMA shall be able to detect compounds of low stability (*e.g.*, formaldehyde) at concentrations as low as 1 ppmv.

R5. MOMA shall investigate the biotic or abiotic origin of organic chiral molecules (*e.g.*, amino acids, carboxylic acids, and amines) by analyzing their enantiomers at molecular concentrations as low as 1 ppmw.

R6. MOMA shall be able to detect light volatile organics (*e.g.*, benzene, alkanes, and amines) at concentrations as low as 1 ppmw.

R7. MOMA shall be able to detect refractory organics (*e.g.*, heavy PAHs, kerogen-like material) with molecular weights up to 1000 u at concentrations as low as 10 ppmw.

R8. MOMA shall be able to provide diagnostic presence/absence information on contextual elemental composition (*e.g.*, inorganic breakdown products produced upon pyrolytic heating or laser desorption).

R9. MOMA shall be able to provide diagnostic presence/absence information on contextual mineralogical composition (*e.g.*, inorganic breakdown products produced upon pyrolytic heating or laser desorption) at a threshold of 1 wt %.

## 4. Instrument Top-Level Description

**FIG. 1.**
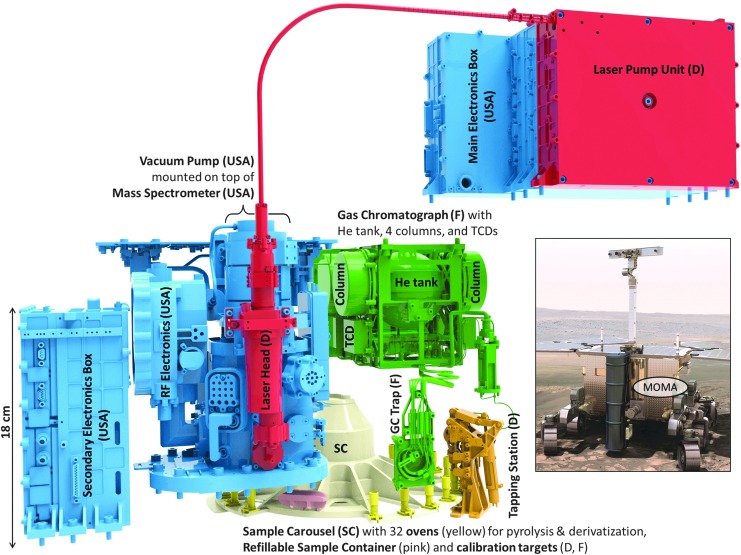
The different parts of MOMA and their contributors (France (F), Germany (D), United States) are displayed in the figure above. The LPU that controls the LH is mounted as a slice on the MEB (top right). Sample carousel (SC) and refillable sample container are not part of MOMA. LH, Laser Head; LPU, Laser Pump Unit; MEB, Main Electronic Box; MOMA, Mars Organic Molecule Analyzer.

**FIG. 2.**
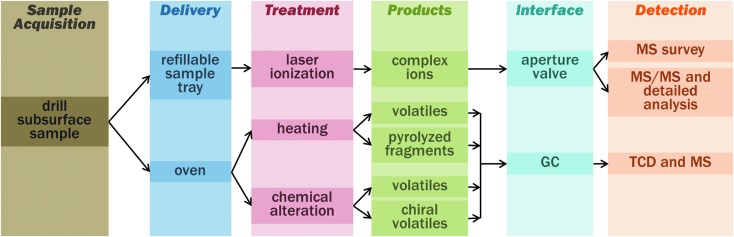
Chain of sample processing and analysis. The subsurface sample is received from drill and crushing station. Important decisional points are the MOMA operational mode, in particular, choose delivery to refillable sample tray/laser ionization versus pyrolysis oven and select chemical alteration (derivatization agent) and GC column. In the latter case, the signal is generally detected by both MS and TCD (Section 6.2.2). GC, Gas Chromatography; MS, Mass Spectrometer; TCD, Thermal Conductivity Detector.

The task of the MOMA instrument is the detection and identification of various molecular species and classes at low concentrations with high analytic specificity. One mode of MOMA is the use of GCMS for volatile molecule characterization. Volatile compounds thermally evolved from solid samples in a pyrolysis oven are separated by the GC and then analyzed individually with the MS. For nonvolatile molecules, MOMA provides a method (LDMS) to produce gas-phase ions by high-intensity laser pulses applied directly to a crushed sample surface. These ions are transferred into the MS and analyzed. Both modes of operation use a common linear ion trap MS (ITMS) for detection and identification of molecular ions. Table 1 summarizes the basic characteristics of the MOMA instrument. Figure 1 shows a three-dimensional (3D) view of the MOMA instrument in its flight configuration, and Figure 2 outlines a decision tree and the flow of events for a notional sample analysis.

**Table 1.** Main Characteristics of the Mars Organic Molecule Analyzer Instrument

*Mass*	*11.5 kg w/o margin*
Average power	65 W (LDMS)
	82 W (GCMS)
Maximum power	133 W (LDMS)
	154 W (GCMS)
Operational temperature range	(−40 + 20)°C (upper value limited by laser)

GCMS = Gas Chromatography–Mass Spectrometry; LDMS = Laser Desorption Mass Spectrometry.

Samples to be investigated will be powdered (crushed) drill cores of sedimentary rocks obtained from the uppermost 2 m of the martian surface. For LDMS, samples are dispensed into a refillable container (Figs. 1 and 2) and leveled, providing a flat sample focal plane for the MOMA laser as well as other optical instruments. The LDMS mode may be used to determine the presence and chemical makeup of nonvolatile compounds in the sample and the results used in subsequent science operation decisions. Second, in GCMS, samples are dispensed into one of the MOMA ovens. There, samples may be directly heated (pyrolyzed) or subjected to a derivatization procedure. The evolved species are transferred to a GC where the compounds are separated into a time sequence and then, after detection by a TCD (thermal conductivity detector), fed into the MS to be separated by mass. The combination of methods covers a wide range of molecules from the light- (*e.g.*, chloromethane) to medium-sized ones (*e.g.*, naphthalene) by GCMS and up to more complicated molecules (*e.g.*, peptides, large PAHs) by LDMS at rather low detection limits (as low as 10 ppbw for GCMS and in the sub-pmol/mm^2^ range for LDMS). In this study, it should be noted that limits of detection are generally given as bulk abundance levels (ppmw, Section 3), while MS testing in the laboratory often requires the use of samples with a known density of these molecules on some substrate (pmol/mm^2^). This discrepancy is unavoidable for technical and practical reasons. However, both quantities (bulk abundance and surface density) can be related to each other through basic assumptions on the accessibility of organic molecules in an inorganic matrix allowing testable quantities to be used in the verification of MOMA's performance as outlined below.

The components of MOMA related to GCMS have strong space heritage dating back to the days of Viking. Most recent developments include the COSAC experiment (COmetary SAmpling and Composition) onboard the comet lander Philae (Goesmann *et al.*, 2007) and SAM onboard Curiosity (Mahaffy *et al.*, 2012) on which several members of the MOMA team were also involved. The LDMS method is of more recent design and the first such implementation developed for Mars exploration. The concept of joining the GCMS and LDMS functions through a common MS and pumping system, identified at the first Pasteur payload meeting in 2004, led to the present tightly integrated MOMA system.

## 5. Instrument Development

### 5.1. Team organization

The MOMA instrument is assembled from modules produced and tested at several institutions. The Rosetta heritage ovens and tapping station (TS) are developed at the Max Planck Institute for Solar System Research (MPS) in Germany. The gas chromatograph (GC) is built at Laboratoire Interuniversitaire des Systèmes Atmosphériques (LISA) and Laboratoire ATmosphères, Milieux, Observations Spatiales (LATMOS) in France with some German participation in the electronics by MPS. Pyrolysis GC testing campaigns have been managed in a partnership between the German and French teams. The MS and its drive electronics, as well as the main electronics of MOMA, are developed by NASA's Goddard Space Flight Center (GSFC) and its partners at the Space Physics Research Laboratory (SPRL) at the University of Michigan in conjunction with Battel Engineering. The laser drive electronics are built at MPS, while the laser head (LH) is designed and built at Laser Zentrum Hannover (LZH).

The integration of MOMA hardware in the rover is distributed as well: MOMA does not form a single compact unit, but remains modular with numerous mechanical and thermal interfaces with the rover's Analytical Laboratory Drawer (ALD). As such, the integration activities are highly collaborative between rover and instrument teams. The sample drilling and distribution system, which needs to retrieve, grind, portion, and deliver the samples to either a refillable container for LDMS or the ovens for GCMS, is not part of MOMA. The carousel element of this system, a critical functional part of the MOMA analytical sequence, but not part of MOMA, is included in Figure 1.

### 5.2. Instrument integration flow

Upon delivery, the MOMA instrument will be integrated into the analytical drawer of the rover (Vago *et al.*, 2017). The integration will happen at Thales Alenia Space in Torino, Italy (TAS-I). The steps to arrive at an integrated instrument are as follows: The ovens and the TS are directly delivered from MPS to TAS-I. They need to be among the cleanest parts of MOMA since they will be in direct contact with the samples and will be situated in the Ultra-Clean Zone (UCZ) (Vago *et al.*, 2017). Hence, they will be delivered in specially designed transport containers that are pressurized to prevent exposure to ambient conditions. The laser electronics are delivered from MPS to LZH to complete the laser. The laser is delivered to GSFC so that the LH can be integrated to the MS while the laser electronics become part of the larger electronic unit built at GSFC. This constitutes the LDMS subsystem of MOMA. The total LDMS is then delivered to TAS-I. The MS itself forms a part of the closeout of the UCZ: the bottom plate and inlet valve system for LDMS are therefore subject to the same extreme contamination controls as the ovens and TS. After necessary interface testing, the GC is also directly delivered from LISA/LATMOS to TAS-I.

### 5.3. Instrument verification

The modularity requires multiple coupling tests between instrument subsystems to verify instrument performance. To date, several GCMS campaigns have been conducted at GSFC (Section 6.2.2.6) where a series of models of the GC were coupled to breadboards and an engineering model of the MS. The sample chain from oven to TS to GC was verified during campaigns at LISA/LATMOS. The LDMS performance was tested with lasers and mass spectrometers at different stages of development as well. Final instrument-level flight testing and calibration under Mars environmental conditions will occur as each subsystem achieves performance verification and acceptance at the component level.

### 5.4. Achieved milestones and deliveries

The Preliminary and Critical Design Reviews (referred to as PDR and CDR) of the MOMA instrument are conducted by ESA. The MOMA PDR was completed in 2016, whereas the MOMA CDR will occur late in 2017. The NASA CDR of the MS subsystem was already completed in September 2014. The structural and thermal model of the instrument was delivered in early 2016. The Qualification Simulator Model (QSM) has been delivered in stages over 2015–2016. This will be followed by the Flight Model (FM) when ready and all delivery reviews have been passed.

## 6. Instrument Subsystems

### 6.1. Oven and tapping station subsystems

Pyrolysis and derivatization implemented in an array of ovens was already developed for the COSAC GCMS onboard Philae (Goesmann *et al.*, 2007). With this heritage, the path of development for specific science requirements and anticipated sample compositions for MOMA was started. For the ovens, a larger diameter was chosen to pyrolyze larger amounts of sample to account for the lower organic concentrations expected in Mars samples. The development ended with ovens of a diameter of 6 mm and an inner volume of slightly more than 200 mm^3^. One aliquot of powderized martian drill sample from the jaw crusher and dosing station (both are part of the SPDS, *i.e.*, the sample processing and distribution system) is expected to have a volume of ∼100 mm^3^ and may correspond to an approximate mass of 100 mg (assuming a bulk density of 1 g/cm^3^). Given the uncertainty on that volume, the oven can only receive a single aliquot from the SPDS. All ovens are designed for *single use*, that is, they shall receive one aliquot of a given sample that may then be subjected to one or several processing steps, for example, two heating cycles in DTA mode (Section 2) or stepwise pyrolysis (Section 7). Pyrolysis and derivatization ovens will be heated to a maximum temperature of 850°C and 600°C (thermochemolysis), respectively (Table 2). The ovens are made from high-temperature steel. Their inner temperature is measured by a Pt100 sensor in the bottom of the oven. The heating is conducted by a platinum filament incorporated in a ceramic cylinder. To minimize thermal losses during heating, the oven is mounted on a thin-walled stand and the oven with the heater is wrapped in platinum foil to decrease radiative losses during heating. The TS ensures the electrical and pneumatic contact with each oven: (1) spring pins in connectors at the oven's base engage to achieve the necessary electrical contact between oven and TS and (2) a TS zirconia sphere is pressed onto the oven top's knife edge for gas sealing. The power lines to the oven heater and the lines to the temperature sensor are contacted by a small bridge that engages with the oven when the oven is closed by the zirconia sphere. The latter provides connection to two gas lines: one delivers helium, while the other line conducts the pyrolysis products entrained in the helium flow to the GC for separation and analysis.

**Table 2.** Characteristics of Mars Organic Molecule Analyzer Ovens (for GCMS) and the Rover's Refillable Sample Container (for LDMS)

Thirty-two GC ovens	Twenty for pyrolysis/EGA, 12 for derivatization
Maximum temperature of GC oven	850°C for pyrolysis/EGA, 600°C for derivatization
Desired sample quantity	100–200 mm^3^ for GC oven ∼600 mm^3^ for refillable sample container

A GC oven can receive any (powdered) sample whose bulk volume (including pore space) is in the range 100–200 mm^3^ (200 mm^3^ is the critical upper limit, especially for pyrolysis ovens).

EGA = Evolved Gas Analysis.

The TS was modified from the Philae design to provide for larger ovens and the difference in electrical contacts. As a result of the redesign, an increase in force of 200 N was necessary to close the oven and resulted in a design optimization effort, thus increasing the number of structural and mechanical parts of the TS considerably. The greatest changes were necessary in the spring system to ensure that the required force could be applied over a short stroke. Given the need to minimize any potential organic contamination within the UCZ (Vago *et al.*, 2017) to meet the strict ExoMars contamination control requirements, several materials were replaced by metallic or ceramic parts.

The concern that a blocked TS would represent a single-point failure for all rover ALD science operations (MOMA-LDMS, MicrOmega, RLS) led to the design of an emergency release mechanism for the oven TS. Several possible emergency releases were evaluated resulting in implementation of a motorized knee leverage release.

#### 6.1.1. Derivatization

To allow analysis by GC, analytes have to be extracted from the mineral matrix into the gas phase. MOMA will accomplish this through thermal desorption (Buch *et al.*, 2009) instead of solvent extraction, which is generally used in laboratory experiments, but requires excessive resources (Buch *et al.*, 2006, 2009). Once released, the volatile, nonreactive, and thermally stable fraction of the sample can be analyzed with GCMS. Some of the compounds targeted by MOMA contain a labile polar functional group (*e.g.*, -OH, -COOH, -NH, -SH, or -PH). We anticipate that these compounds could form intramolecular bonding that (under the MOMA GC conditions) would result in poor separation or a degraded peak shape, limiting selectivity and/or sensitivity of GCMS for certain compounds. Polar compounds, in particular, tend to be adsorbed on the active surface of the column and on the tubing walls (*e.g.*, transfer lines), which can lead to a peak tailing and poor specificity. Given the importance of polar compounds in the characterization of biosignatures on Mars, these difficulties drive MOMA's requirements on heated line temperatures and onboard derivatization chemistry.

In practice, a derivatization agent produces a controlled reaction to protect the reactive group, thus decreasing the polarity of the molecule and thermally stabilizing it (Knapp, 1979). Several derivatization agents were considered and reagents were selected based on chemistry of the targeted labile group and on the required yield of the derivatization reaction. In addition, the nature of the detector used for the analysis is a key parameter to be considered. Two derivatization reagents have been chosen for implementation in MOMA: *N,N*-methyl-tert-butyl-dimethylsilyltrifluoroacetamide (MTBSTFA) and *N,N*-Dimethylformamide dimethyl acetal (DMF-DMA).

##### 6.1.1.1. MTBSTFA

**FIG. 3.**
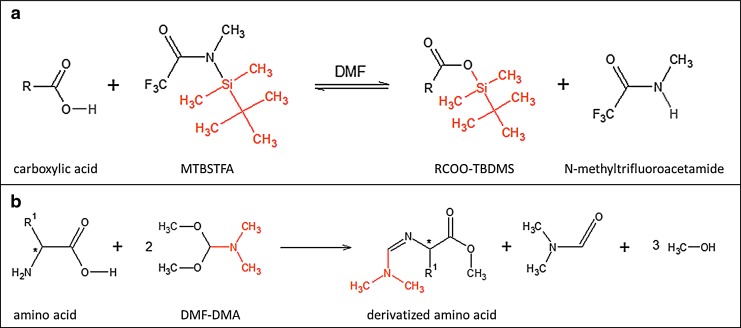
**(a)** MTBSTFA/DMF derivatization reaction with a carboxylic acid. **(b)** DMF-DMA derivatization reaction with an amino acid. The asymmetric center (*) is conserved.

MTBSTFA is a silyl reagent that readily reacts with a wide range of labile groups by replacing labile hydrogen by a tert-butyl-dimethylsilyl group (Si(CH_3_)_2_C(CH_3_)_3_) (TBDMS), and the yield of this reaction is close to 96% (Mawhinney and Madson, 1982). The resulting derivatized compound is sufficiently stable to enable GC separation and sensitive detection by mass spectrometry. Moreover, when using mass spectrometry as the detection technique, the ions produced from silylated species generate very characteristic ions, making their identification easier, and the MS response is enhanced due to the presence of fluorine in the ions. Usually, *N,N*-dimethyl-formamide (DMF) is added to MTBSTFA not only to play the role of a solvent but it can also promote the derivatization reaction as a proton acceptor. A disadvantage of MTBSTFA is its preferential reaction with water to produce both mono- and bisilylated water compounds that can produce sizable background signals and compete with derivatization of the compounds of interest, as shown with measurements currently done with the SAM experiment on Mars (Leshin *et al.*, 2013). Moreover, no separation of enantiomers derivatized when using this technique has ever been obtained by our team, nor has it ever been reported in the literature. While the reason for this lack of chromatographic separation is unclear, MTBSTFA cannot be used for enantiomer analysis. MTBSTFA reactions are typically conducted in the laboratory (a typical example is shown in Fig. 3a) at a temperature of ∼75°C, but for *in situ* analysis on Mars, we anticipate thermal desorption of organic matter to occur at a higher temperature, in the vicinity of 250°C, and silyl derivatization with MTBSTFA is therefore implemented on MOMA at 250°C (the sample shall be held at that temperature for about 10 min). At this temperature and in the absence of oxygen, MTBSTFA has been shown to be stable for more than 2 h.

##### 6.1.1.2. DMF-DMA

DMF-DMA is a methylation reagent developed for use by COSAC that reacts solely with fatty acids, primary amines, and amino acids, replacing a labile hydrogen atom by a methyl group (Meierhenrich *et al.*, 2001). Although it is less efficient than MTBSTFA (detection limit of 1.3 ppm rather than 80 ppb for amino acids), it is still compatible with the concentration of organic compounds found in micrometeorites due to a better MS response. The most important property of DMF-DMA is its capability to enable separation of amino acid enantiomers when used with an enantioselective GC column (Freissinet *et al.*, 2010). With MOMA's enantioselective column (Table 3), DMF-DMA derivatization GCMS will allow us to quantify robustly and sensitively the presence of an ee. To avoid racemization, the DMF-DMA methylation reaction (typical example in Fig. 3b) is programmed to occur at 145°C (temperature of the capsule eutectic) for 3 min (Freissinet *et al.*, 2010). In this way, we perform a single-step extraction and derivatization of labile (chiral and nonchiral) compounds.

**Table 3.** List of GC Columns Selected for Mars Organic Molecule Analyzer GC and Their Main Characteristics

*Name (supplier)*	*Stationary phase*	*Dimensions L/ID/d_f_ (m/mm/μm)*	*Compounds targeted*
MXT Q BOND (Restek)	Divinylbenzene	25/0.25/8	Inorganic volatile molecules C1-C5 organic molecules
MXT CLP (Restek)	Not available from the supplier	25/0.25/0.25	C4-C25 organic molecules
MXT 5 (Restek)	95% dimethylsiloxane	25/0.25/0.25	C4-C25 organic molecules
	5% diphenylsiloxane		
CP Chirasil Dex CB (Agilent)	enantioselective, B-cyclodextrin bonded to dimethylpolysiloxane	25/0.25/0.25	Organic enantiomers

#### 6.1.2. Thermochemolysis

While GC of many nonvolatile compounds in space is enabled by derivatization (MTBSTFA and DMF-DMA), the moderate reaction temperature range of 75–300°C does limit access to refractory and/or insoluble organic material, including macromolecular organics such as kerogen-like compounds. Pyrolysis of such organic matter occurs at much higher temperatures (800–1000°C) and results in typically unacceptable fragmentation of the parent molecule due to thermal stress, limiting important identifications. That is the reason why several workers (Challinor, 1989, 2001; Rodier *et al.*, 2005) have introduced thermochemolysis, which is a thermally assisted hydrolysis/methylation technique. It allows the analysis of refractory and/or insoluble matter in complex matrices containing low amounts of organic material with minimal destruction of the organic material (Geffroy-Rodier *et al.*, 2009). Thermochemolysis is a technique that combines the advantage of derivatization (methylation) without the drawbacks of pyrolysis. Indeed, thermochemolysis with tetramethylammonium hydroxide (TMAH) improves the pyrolysis technique by mild thermal decomposition (hold sample at 600°C for 40 s) and a selective cleavage of ester and ether bonds rather than random and noncontrolled thermal decomposition and fragmentation of the organic material. TMAH thermochemolysis is also included in a small number of sample cups available in the SAM investigation (Mahaffy *et al.*, 2012).

**FIG. 4.**
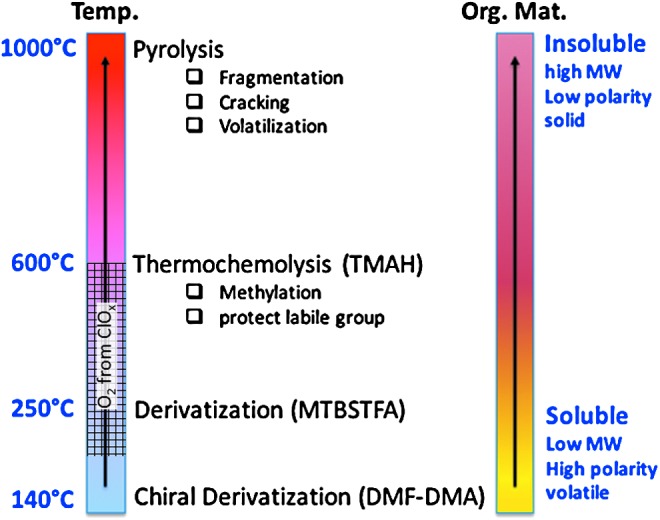
Overview of the space-compatible sample treatment for *in situ* GCMS analysis. Release of oxygen and chlorine from oxychlorine species in the temperature range 200–600°C (Archer *et al.*, 2016) competes with MTBSTFA derivatization and thermochemolysis. GCMS, Gas Chromatography–Mass Spectrometry.

Figure 4 demonstrates the complementarity of thermochemolysis with desorption/derivatization (MTBSTFA and DMF-DMA) and pyrolysis at, respectively, lower and higher temperatures. However, this experimental strategy is jeopardized by the competing combustion of organic compounds by oxygen that is released during decomposition of oxychlorine species (up to ∼1 wt %, Hecht *et al.*, 2009) in the sample. SAM onboard the Curiosity rover uses oxygen detected during EGA experiments as a proxy for the presence of oxychlorine species in soils and sediments (Glavin *et al.*, 2013; Archer *et al.*, 2016). Based on SAM-EGA data, the oxygen is generally released in a 100–150°C-wide temperature window that can move up and down within ∼200°C and ∼600°C, as indicated in Figure 4. Moreover, these data indicate that the abundance levels of oxychlorines in Gale surface samples vary by at least one order of magnitude (Archer *et al.*, 2016). Thus, clues from different instruments (including MOMA itself) must be used to assess the potential presence and abundance of oxychlorine species in drill samples (Section 7.3).

#### 6.1.3. Wet chemistry: storage and release of chemical agents

**FIG. 5.**
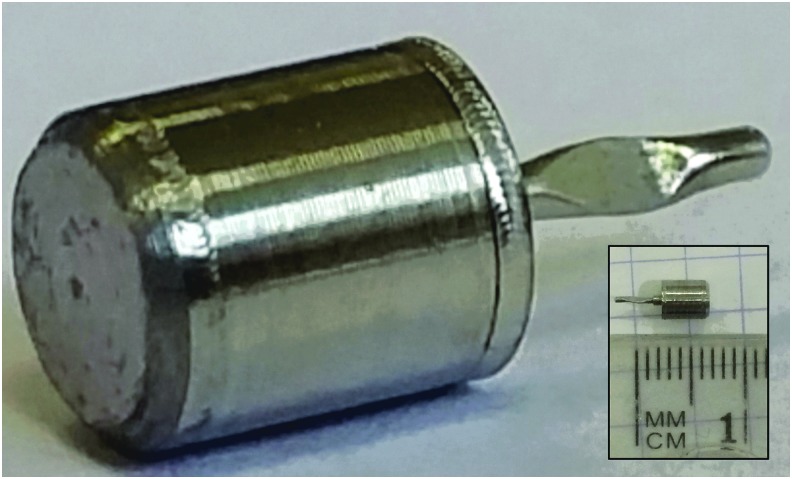
Picture of an MOMA derivatization capsule. The inset shows the same capsule next to a metric length scale (the smallest subdivision is 1 mm). The cylindrical container is 4.3 mm high and 3.2 mm in diameter (external dimensions). The 4-mm-long shaft (in the right part of the image) is the remaining part of the filling port.

To be adapted for use in space, each chemical reagent must be stored under a robust hermetic seal to avoid any potential background contamination of the chromatograms. Moreover, as described in Figure 4, each wet chemistry technique occurs at different temperatures, from 140°C for the chiral derivatization up to a maximum of 600°C for thermochemolysis. To accommodate this range, each reagent has been sealed into a stainless steel capsule. The stainless steel surface has been treated with a Silcosteel^®^ passivating layer to avoid any oxidative attack by the corrosive reagents (MTBSTFA, DMF-DMA, and TMAH). The Silcosteel coating is thermally resistant up to 400°C. The capsule has the following dimensions: external *φ* = 3.2 mm, h = 4.3 mm, with an internal volume of about 25 μL. Because each reagent has to be released at a specific temperature, three different eutectic alloys have been implemented to seal the capsule up to a minimal temperature reached by the oven (Fig. 5). Thus, MOMA capsules will release DMF-DMA at 145°C, MTBSTFA/DMF at 221°C, and TMAH at 309°C. The reagent is initially loaded into capsules through fine tubing, which is hermetically closed by using a seal welding technique. About 15 μL of each reagent is stored in each capsule.

### 6.2. Gas chromatograph subsystem

Considering the number of organic and inorganic volatile compounds, including isomers, which potentially could be released from a martian sample, mass spectrometry alone is not sufficient to discriminate and identify all molecules. Moreover, mass spectral techniques do not offer the possibility to separate and quantify the relative amounts of enantiomers. For MOMA, a GC was chosen to achieve the requisite additional separation, following the approach used on the Viking landers/GCMS (Biemann *et al.*, 1977) and on MSL/SAM (Mahaffy *et al.*, 2012). A description of the GC instrument, the basic way it operates, and its current performance is provided here.

#### 6.2.1. Design requirements

To meet the MOMA science objectives (Section 3), the GC must be able to separate a wide range of organic compounds. The main classes of chemical species to be separated are (i) regular volatile organic molecules consisting of 1 to about 25 carbon atoms; (ii) organic enantiomers; (iii) species produced from derivatization with the three chemical reactants to be used in MOMA (Section 6.1); and (iv) inorganic volatiles. The GC therefore contains four individual column modules, each targeting a specific class of chemical compounds, packaged into a highly constrained instrument volume.

#### 6.2.2. Design overview

##### 6.2.2.1. General architecture

**FIG. 6.**
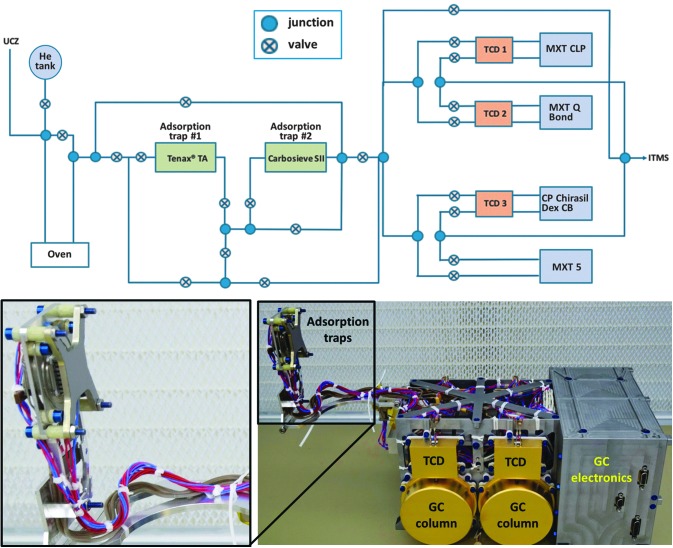
Top: Simplified gas flow diagram of MOMA GC. Volatiles derived from the sample are carried by a steady helium flow, trapped, and concentrated in one of the adsorption traps and thermally released in backflush mode. Finally, the volatiles are separated by one of the GC columns (MXT CLP, MXT Q Bond, CP Chirasil Dex CB, or MXT 5) and detected by both TCD and ITMS, except in the case of MXT 5, where the separated compounds are detected by ITMS only. Bottom: GC QSM as of March 2016 with zoom of the adsorption traps. ITMS, Ion Trap Mass Spectrometer; QSM, Qualification Simulator Model.

The MOMA GC (Fig. 6) is packaged into a volume of 14 × 12 × 20 cm and a mass of 1.6 kg. It provides all necessary functionality for GC, including injection, separation, detection, carrier gas delivery and flow regulation, fluidic plumbing, and control electronics.

##### 6.2.2.2. Injection system

To get the best separation of the analyzed compounds, their injection into the separation system (chromatographic columns) should be as brief as possible, generally a couple of seconds in laboratory applications. Direct transfer to a column for the low pyrolysis heating rates of the MOMA ovens (∼200°C/min) would result in poor chromatographic resolution and would compromise the analytical capabilities of the GC. For this reason, adsorption/thermal desorption traps are used on MOMA.

The MOMA design uses two separate complementary traps, each devoted to concentration and injection of a specific range of compounds. Taking into account the chemical species to be analyzed with MOMA, Tenax^®^ GR adsorbent is used to trap most of the organic compounds consisting of more than three carbon atoms, whereas Carbosieve SIII is used for the lightest organic molecules and inorganic volatiles. For each trap, a few tens of milligrams of adsorbent are packed into a 3-cm-long stainless steel tubing with an internal diameter of 1.6 mm.

Temperature control of the traps is provided by a Peltier cooler, holding the traps at ∼30°C below the experiment ambient temperature. Targeted volatiles are trapped from the gas evolved from the oven while the trap is cooled to enhance adsorption. Once trapping is done, a dedicated heater brazed to the tubing rapidly raises trap temperatures to 300°C in less than 15 s. This near-flash heating quickly releases the trapped chemical species to the chromatographic columns to enable a sufficiently short injection time. To further improve the recovery efficiency from the trap, a backflush procedure is used by reversing the helium flow during the desorption step. The gas flow pathway was designed to allow maximum flexibility for injecting the trapped species. While only one trap may be used at a time, each trap can be coupled to any of the four GC columns (eight available analytical combinations).

##### 6.2.2.3. Separation of volatiles

MOMA includes four columns (Table 3) allowing complementary separation of different compound classes. The 25-m-long × 0.25 mm internal diameter columns are of high chromatographic efficiency (more than 25,000 theoretical plates) while meeting overall resource constraints. Stainless steel column tubing was selected for its robustness and ease of integration with gas plumbing. Each column is integrated into a module (Fig. 6), coiled around a cylinder with a dedicated heater to control the column temperature from ambient to 250°C (for CP Chirasil Dex CB (Table 3), the temperature may not exceed 220°C). Both column and heater are imbedded in ceramic glue to ensure robustness and temperature homogeneity of a whole column. A gold-coated cap covers each module for mechanical packaging and thermal control reasons.

##### 6.2.2.4. Detection of volatiles

The MS (Section 6.4) is used to record the retention time of each compound eluting from the column over a period of 30 min following the injection (Section 6.2.2.2). Three of the four chromatographic columns include a TCD that can also measure the retention time of the eluted compounds (Fig. 6 and Table 3). MXT-5, the most versatile of these four chromatographic columns, is not configured with a TCD for risk mitigation in the event of TCD failure. These TCDs are based on compact micro-electromechanical systems technology with picomole sensitivity. The power requirements of the TCDs are low and their use allows acquisition of a signal with sufficiently fast temporal sampling for peak resolution. In spite of a lower sensitivity compared with the MS, the linearity of the TCD response will complement the MS measurement to quantify the most abundant compounds released by the samples.

The TCDs are accommodated on a dedicated plate, which is mounted to ensure thermal decoupling between the chromatographic column and the TCD and to provide the 110°C required by the detectors. However, this operating temperature may present a cold trap for compounds eluting from the columns. For this reason, no TCD is coupled to the MXT5 column to limit the presence of cold spots in this channel and enable the analysis of more complex organic species that could condense when analyzed with the other modules.

##### 6.2.2.5. Gas handling system

The fluidic system of the GC comprises a Gas Tank (GT), one pressure control valve (PCV), 19 on/off valves allowing the selection of different fluidic pathways by a series of valve configurations, and stainless steel tubing (with external/internal diameter of 0.8/0.5 mm) used as plumbing between components (see Fig. 6 for a simplified diagram).

The GT contains the carrier gas required for chromatographic analyses. The carrier gas selected is ultrapure helium (99.9999%) as it is the most efficient carrier gas for chromatography (after H_2_) and is safe to handle. The GT is spherical (8 cm in diameter) and made of titanium. It contains ∼150 mL of helium, initially at a pressure of 80 bars.

The PCV allows regulation of carrier gas column inlet pressure, and subsequently the carrier gas flow rate through the column. The pressure can be set from the ambient to about four bars absolute pressure and is controlled by physical measurement of the inlet pressure with a micropressure sensor mounted just after the PCV. A control pressure sensor is also mounted just before the column to get the exact column inlet pressure, which is a key parameter to achieve good separation and to help when analyzing the GC data.

Except for the GT, all components of the GC fluidic system are heated up to ≥135°C (the maximum temperature is controlled by convection and available power) by different heaters of various shapes, depending on the component. The upper mass limit of detectable organic compounds depends critically on the type of the chromatographic column and its temperature ramp (Section 6.2.2.4), as well as the heating of the gas transfer tubing. Overall, the upper mass limit is expected to lie around 300 and 500 u for, respectively, pyrolysis and derivatization runs.

##### 6.2.2.6. GCMS coupling campaigns

To demonstrate the functionality of the GCMS experiment, the two independent instruments need to be coupled and their performance characterized as a single unit. The coupling campaigns are international efforts and therefore finite-time opportunities to test the interface of hardware, electronics, and software controls at a given point in time for each development effort.

The GC used for the campaign was the QM (qualification model), comprising a hydrocarbon trap as the injector, an MXT-5 column (25 m, 0.25 mm, 0.25 μm, Restek), and a TCD. The GC was coupled with ETU (engineering test unit) of the ITMS. The TCD and analytical column can be heated and their maximum temperature is, respectively, 100°C and 200°C. Ultrahigh-purity helium (99.9999%) is used as the carrier gas at a mean 1.1 mL/min flow rate.

In this experiment, volatile compounds contained in a gas mixture (butane, pentane, hexane, and benzene, 1000 ppm each) were used as calibrant standards. A sampling loop of 10 μL allowed for the injection (via an atmospheric inlet) of a known quantity of analyte onto the Tenax GR trap. The gas mixture was trapped by the Tenax trap and released at 156°C. The column temperature was kept constant at 30°C.

**FIG. 7.**
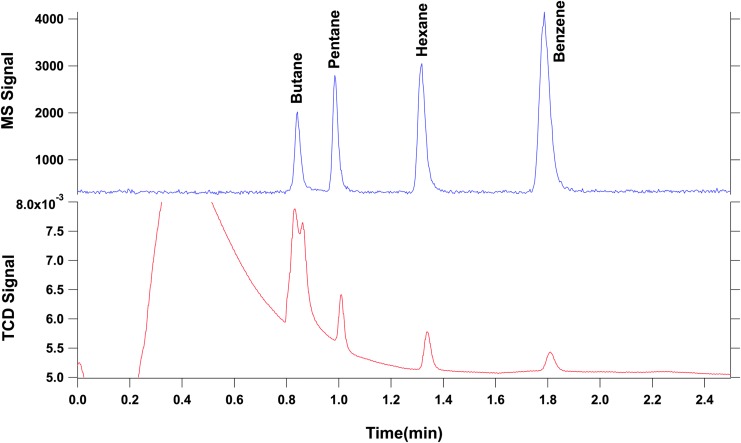
Separation of volatile compounds (butane, pentane, hexane, and benzene). The chromatogram obtained shows good separation of the four compounds injected in both the MS (above) and TCD (below) signals. The double peak observed on the TCD signal at a retention time of 0.85 min is due to the presence of air, which is not observed by the MS as it is below its mass range. MS, Mass Spectrometer.

**FIG. 8.**
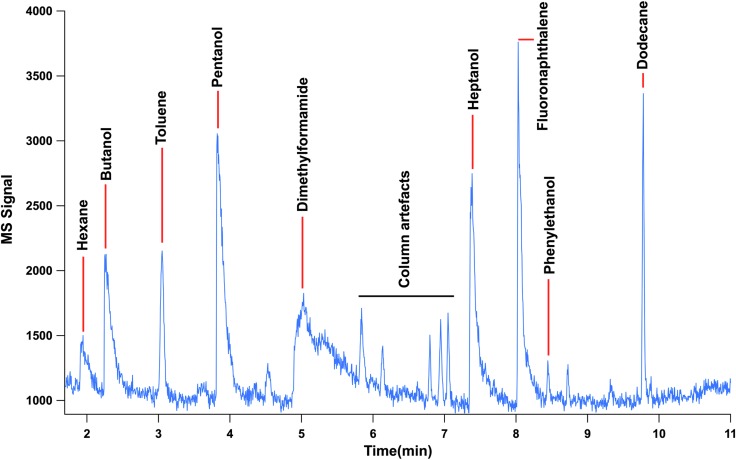
MS chromatogram for separation of the mixture of phenylethanol, 1-butanol, methyl-acetate, hexane, benzene, toluene, dodecane, heptanol, pentanol, fluoronaphthalene, and dimethylformamide diluted in methanol. Methyl acetate and methanol were not detected by the MS since these molecules (or their heaviest fragments) have masses below its mass cutoff set to *m/z* ∼ 40.

A second liquid calibration mixture was used for injection into the MOMA oven and comprised 5 μL of phenylethanol, 1-butanol, methyl-acetate, hexane, benzene, toluene, dodecane, heptanol, pentanol, fluoronaphthalene, and DMF diluted in 100 μL of pure methanol. For the liquid mixture, a volume varying from 0.05 to 0.1 μL was injected in the MOMA oven at ambient temperature. The oven was sealed and heated to 350°C under helium flow and volatiles were trapped by the Tenax GR adsorbent. Finally, the Tenax trap was rapidly heated up to 300°C and the volatilized sample was injected in the GC column. The column temperature was ramped from 30°C to 200°C with a ramp rate of 15°C/min. Figures 7 and 8 show the signals acquired during both experiments.

In conclusion, we successfully demonstrated the first end-to-end GCMS coupling tests with a QM GC (including high-fidelity oven) and ETU MS. Gaseous and volatile calibration compounds were successfully separated and detected in GC-TCD mode and in GCMS mode as well. Limits of detection have been determined and a value under the pmol/mm^2^ level has been obtained for each analyzed compound, as required for MOMA.

### 6.3. Laser subsystem

The laser subsystem serves as the ionization source in the LDMS mode of operation. It has been designed to efficiently desorb and ionize primarily nonvolatile, high-molecular-weight organic molecules in the crushed rock samples delivered by the SPDS, while minimizing thermal and photochemical alteration/degradation effects on the sample.

#### 6.3.1. Design requirements

MS subsystem (LDMS mode) requirements (Section 6.4.1) are the primary drivers for the design of the laser subsystem. Specifically, a short pulse width (τ_P_ ≈ 1 ns) UV (λ = 266 nm) laser was selected due to its efficiency in desorbing and ionizing organic molecules and thus enabling their sensitive mass spectrometric detection. Aromatic compounds, in particular, are readily ionized by the laser as they exhibit strong absorption near 266 nm wavelength. The laser is incident onto the sample at 44° relative to surface normal and is focused to ≈400-μm-diameter (slightly elliptical) spot on the sample surface. The laser pulse energy on the FM laser is expected to be adjustable from 13 to 130 μJ, which approximately translates into peak beam irradiance of 35 to 250 MW/cm^2^. The laser is capable of emitting bursts of up to 50 laser pulses at a 100 Hz repetition rate, within a thermally limited average repetition rate of 2 Hz. The tunability of both the average pulse energy and the number of laser pulses within a burst is critical in ensuring that an appropriate amount of ions is injected into the ion trap, preserving optimal mass spectral performance of the MS subsystem (see LDMS mode automatic gain control description in Section 7.4). A detailed list of laser subsystem requirements is given in Table 4.

**Table 4.** Performance Requirements for the Laser Subsystem

*Specification*	*MOMA laser*
Output wavelength	266 nm
Pulse energy	13–130 μJ
Pulse duration (FWHM)	<2.5 ns
Beam quality	M^2^ <4
Burst repetition rate	100 Hz
Burst laser shots	≤50
Avg. repetition rate	2 Hz
Spot size (on sample)	≈ 400 μm
Peak irradiance (on sample)	>30 MW/cm^2^
Peak fluence (on sample)	>60 mJ/cm^2^
Lifetime	10 Mshot
Operational T	−50°C to +25°C

MOMA = Mars Organic Molecule Analyzer.

#### 6.3.2. Laser architecture

The laser subsystem comprises the Laser Pump Unit (LPU) and the LH. The LPU is mounted on the main electronic box (MEB; Section 6.4.4) and contains all laser electronics as well as the pump diode outputting 70 W peak optical power for ∼140–200 μs at the center wavelength of λ = 806 nm. The pump diode output is fiber-optically coupled into the LH mounted on the side of the MS housing (Fig. 1). It pumps an Nd:Cr:YAG crystal within a compact passively Q-switched oscillator cavity in the LH that emits ≈1.5 mJ of λ = 1064 nm laser pulses with a pulse duration of τ_P_ ≈ 1–2 ns. The output pulses are frequency quadrupled to λ = 266 nm by two frequency conversion stages, spectrally filtered by dichroic mirrors, and a small fraction of the UV output sampled by a calibrated internal photodiode, thus enabling energy monitoring on a pulse-by-pulse basis during LDMS operations. The balance of the UV beam is sent onto the sample located in the UCZ through an external beam steering unit and a Brewster-angled laser window, which hermetically seals the rover's UCZ, yet provides optical access. A slow-focusing optic in the LH ensures the projected beam spot size of ≈400 μm on the sample and sufficiently large focal depth to accommodate a crushed and leveled sample surface expected to exhibit a typical surface roughness of <200 μm. The output pulse energy is adjustable down to 10% of the maximum output energy and is achieved through thermal tuning of the frequency conversion crystals. More detailed information on the MOMA laser optical layout and design can be found elsewhere (Kolleck *et al.*, 2010). Overall, laser/optomechanical design is capable of withstanding mission's shock and vibration requirements as well as maintaining laser to MS ion inlet tube axis alignment (Section 6.4.2.4) within ∼0.5 mm, driven by the tolerance of the ion sampling off the MS inlet axis.

#### 6.3.3. Verification data

**FIG. 9.**
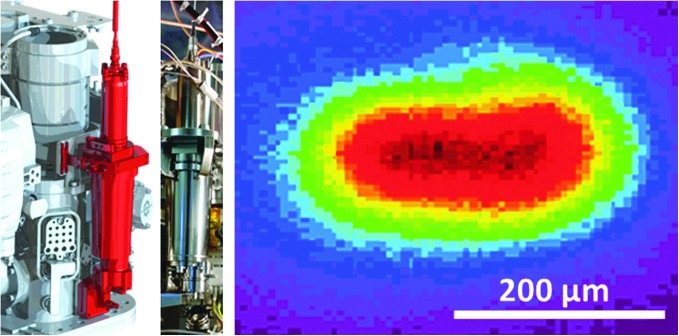
Left: MOMA laser subsystem highlighted in red: LH mounted to the side of the MOMA-MS housing (Fig. 1). Middle: Photograph of the ETU LH and (right) UV beam profile at the sample location for high-energy output of the ETU laser. ETU, Engineering Test Unit.

A flight-like ETU laser subsystem (Fig. 9) has undergone extensive characterization and verification process in both a stand-alone configuration and mounted to the MS FM. Temperature tuning of the frequency conversion crystals has been demonstrated to vary the UV output energy of the ETU laser from 21 to 260 μJ, yielding peak irradiance at the sample location ranging from 50 to 450 MW/cm^2^. A representative beam profile projected 44° onto the sample location for the *highest* output pulse energy is shown in Figure 9 and indicates a full-width tenth-maximum spot size of ≈400 × 200 μm at the sample.

The FM laser is expected to inherit most performance characteristics of the ETU laser, with the largest difference being the output derating to 35–250 MW/cm^2^ due to intense ion generation from most realistic samples when laser intensities exceed ≈250 MW/cm^2^.

### 6.4. Mass spectrometer subsystem

The MS subsystem of the MOMA instrument serves to collect, manipulate, and detect ionized particles based on their respective mass-to-charge (*m/z*) ratios. As described above, the MOMA-MS subsystem supports two independent, but highly complementary, modes of operation: (i) GCMS of gases derived from the pyrolysis of crushed rock powder and (ii) LDMS of sample fines deposited into a refillable tray. Multiple copies of the MS subsystem have been designed, built, and tested with progressive technical maturity, beginning with proof-of-concept prototypes, advancing to the ETU, and culminating in the actual FM and viable Flight Spare (FS). A brief introduction to the driving requirements and basic design of this subsystem is described here; more details can be found in the work by Arevalo *et al.* (2015).

#### 6.4.1. Design requirements

Derived from the original Level 1 Science Objectives of the ExoMars rover mission, a number of specific functional requirements were flowed down to the MS subsystem. Of these requirements, which define key characteristics of the instrument such as acceptable/unacceptable vibration resonances, shock responses, and thermal limitations, the driving requirements for both modes of operation are provided in Table 5. Because each mode of operation targets a specific category of biosignature (although with some overlap), each mode carries distinct performance requirements, such as mass range, sensitivity (limit of detection), and mass resolution. These metrics must be verified experimentally through testing of the FM (and FS) across a range of pressures and temperatures representative of those likely to be encountered on the martian surface.

**Table 5.** Performance Requirements of MOMA-MS Subsystem

*Specification*	*Pyr/GCMS mode*	*LDMS mode*
Targeted biosignatures	Volatile organics (*e.g.*, alkanes, amines, alcohols, carboxylic acids)	Nonvolatile organics (*e.g.*, macromolecular carbon, proteins), inorganic species
Mass range	50–500 u	50–1000 u
Limit of detection	≤nmol analyte^a^ (SNR^c^ > 10)	≤pmol mm^−2^ analyte^b^ (SNR^c^ ≥3)
Resolution (FWHM^d^)	≤1 u	≤2 u
Single scan dynamic range	≥100 over 50–1000 u	
Mass isolation	±5 u precision over 50–1000 u	
Accuracy	Observed mass ±0.4 u actual mass	
Instrument drift	<0.4 u over 30 min	
Operational P	4 to 8 torr of Mars atmosphere	
Operational T	−40°C to +20°C	
Nonop T	−50°C to +60°C	
Survival T	Up to +145°C	

^a^ pyr/GCMS analyte: PFTBA.

^b^ LDMS analytes: Rhodamine 6G and Angiotensin II.

^c^ SNR of mass peak.

^d^ FWHM of mass peak.

FWHM = Full-Width Half Maximum; PFTBA = perfluorotributylamine; SNR = Signal-to-Noise Ratio.

#### 6.4.2. Analyzer architecture

To realize the science objectives of the mission, particularly the characterization of martian (near-)surface materials through LDMS, the MOMA instrument centers around an ion trap mass analyzer. Quadrupole Mass Spectrometers (QMSs), such as those flown to the moon (via LADEE), Mars (via Curiosity rover and MAVEN), and Saturn (via Cassini–Huygens), require high vacuum to achieve performance (<10^−6^ torr). In contrast, ion traps thrive at higher operating pressures (∼10^−3^ torr); thus, this class of analyzer more easily accommodates LDMS operations at Mars ambient pressures. In addition, the MOMA ion trap is 5× smaller in volume than the QMS onboard Curiosity and more than 2.5× smaller in volume than the miniaturized QMS onboard LADEE, MAVEN, and Cassini–Huygens. Because ion traps inherently serve as ion storage devices, the MOMA MS is also capable of conducting advanced ion manipulation experiments, such as SWIFT (Stored Waveform Inverse Fourier Transform) ion excitation and tandem Mass Spectrometry (MS/MS), both techniques are described further below.

##### 6.4.2.1. Linear Ion Trap assembly

In contrast to a traditional Paul ion trap (or 3D type), a Linear Ion Trap (LIT, or two-dimensional type) offers two symmetrical ion injection pathways along the long axis of the rod assembly, thus reducing space-charge effects and enhancing trapping efficiency. As a consequence, the MOMA MS employs an LIT analyzer to directly support both GCMS and LDMS modes of operation. The MOMA-MS LIT comprises four hyperbolic rods situated in parallel with one another, two solid and two with slits down the lengths of their vertices, and two end plates. Defined by an r_0_ = 3 mm (the distance between the solid rods and the central axis of the rod assembly), the MOMA-MS dual-source LIT is a miniaturized (1:4 scale by volume) version of the Thermo Fisher LTQ design. Compared with a Paul trap with a similar physical volume, the LIT leveraged by MOMA offers the following analytical advantages: i) increased ion storage volume and, by extension, reduced space-charge effects; ii) higher trapping efficiency (increased sensitivity); and iii) redundant detection subassemblies as ions are ejected radially through two slit rods. Moreover, the mechanical design and implementation of the MOMA LIT draw strongly from heritage QMS rod assemblies, which are bound by comparable engineering requirements, machining tolerance levels, and assembly logistics.

##### 6.4.2.2. Detection channels

As outlined above, the MOMA LIT ejects ions radially during mass scanning, allowing two detection channels to be situated around the analyzer; these channels may be activated simultaneously to maximize sensitivity or individually as fully redundant elements (thereby doubling the operational lifetime of the instrument). Each detection assembly consists of a stainless steel conversion dynode, which converts ions ejected from the LIT into electrons, and a Channel Electron Multiplier (CEM, Photonis Spiraltron 4219) that collects the electrons generated by the dynode and amplifies them into a detectable current pulse. Both detection chains are shielded from stray electrons and ions to establish low noise floor and improve limits of detection.

The potential of each CEM may be varied from −1.8 to −3.0 kV to maximize electron gain over the life of the device, but the dynode potential is held constant at −5 kV. The high-voltage stability of each detection channel was verified at the operational pressures of the LIT (∼10^−3^ torr). The detection system has been shown to have a maximum count rate of 10^6^–10^7^ counts per second.

##### 6.4.2.3. Electron Ionization Source

When neutral effluent derived from the GC is introduced into the MOMA MS, the gas is ionized by an Electron Ionization Source (EI) with redundant electron gun assemblies. This source is a near-replica of the closed EI sources flown on LADEE/MSL/MAVEN. Each fully operational electron gun relies on a 0.003" diameter W:Re (97:3) filament to generate 10–100 uA of emission with an average electron energy of 70 eV. Each filament is accompanied by a series of ion lenses that serve to focus and direct the beam of electrons toward the neutral gas. An independent stack of lenses accelerates the ion products into the LIT for mass analysis. During mass scanning, the electron beam is gated off by switching the applied voltage on two of the electron gun lenses.

##### 6.4.2.4. Fast-actuating aperture valve

A pull-type aperture valve, inspired by the discontinuous atmospheric pressure inlet valve design pioneered by researchers at the University of Purdue, was developed specifically for the MOMA instrument (Gao *et al.*, 2008). Coupled with the high-pressure operation of the LIT, fast actuation (<20 ms to open or close) of this solenoid-driven valve enables LDMS operations at Mars ambient pressures (*i.e.*, 4–8 torr, primarily CO_2_). Specifically, ions enter the instrument and are trapped at an elevated pressure in the 10s of mtorr. This pressure serves to help cool the ions and promotes very efficient ion trapping. After the aperture valve closes, the Wide-Range Pump (WRP; Section 6.4.2.6) evacuates the extra gas in the MS housing down to a total pressure <0.5 mtorr. This low-pressure environment is both safe to turn on the CEM and dynode high voltages and ideal for collecting a high-quality mass spectrum. As a result of this experimental sequence, the MOMA instrument circumvents the need to hold the sample at vacuum, saving considerable mass, volume, and power that would have been required to implement a vacuum seal.

The aperture valve supports vacuum during clean-up mode bakes, ensures constant pressure during pyr/GCMS testing, and facilitates the dynamics of ion transport (from the irradiated sample surface into the LIT) and rapid pump down during LDMS operations. A sliding ball creates a vacuum-tight seal when compressed against a stationary seat in the valve, which is designed to fail in the closed position as an operational safety. The ETU version of the MOMA aperture valve demonstrated an operational lifetime well in excess of 30,000 actuations while ensuring a leak rate of ≤10^−6^ mbar l/s of helium.

##### 6.4.2.5. Micropirani pressure sensor

Because the MOMA MS operates at fundamentally higher pressures than heritage QMS instruments, a micropirani pressure gauge (based on a commercial Heimann transducer) was qualified for the ExoMars mission. This device, defined by two temperature- and pressure-sensitive resistors contained within shared silicon housing, operates at pressures ranging from 10^0^ to 10^−6^ torr and consumes on the order of ∼0.5 mW (Völklein *et al.*, 2013). Although the response time of the gauge is limited by its heat capacity, which dictates the time required for the resistors to reach equilibrium, a physics-based algorithm enables the sensor to accurately predict pressures under dynamic conditions associated with LDMS operation at Mars ambient pressures. Pressure changes with time constants of ≤0.25 s have been tracked down to 0.5 mtorr with a relative error of ≤3.4% (2σ [Southard *et al.*, 2014]).

##### 6.4.2.6. Wide-Range Pump

The MOMA WRP employs the same core design as the two 100 krpm WRPs now operating successfully on Mars aboard the SAM suite on MSL. The WRP consists of a molecular drag pump in series with a turbomolecular pump designed to exhaust to ambient Mars pressures (qualified for the range 4 to 8 torr). MOMA utilizes a single WRP with a pumping speed of ∼4 L/s and a compression ratio of 10^8^ for N_2_ to provide (1) consistent pumping efficiency against a constant helium flow for GCMS mode and (2) a dynamic pump down environment for LDMS mode.

#### 6.4.3. Temperature control

The MOMA instrument resides within a protective cavity held at Mars ambient pressures in the rover's ALD, thus heat transfer is dominated by thermal conduction rather than forced convection. As a result, the instrument relies on mechanical contact with the upper and lower decks of the ALD for thermal control. With an array of heaters and thermal straps, the MS subsystem employs four thermal zones to control the temperatures of the sample path and critical hardware elements, including the manifolds (Zone 1); ion trap and mechanical housing (Zone 2); WRP (Zone 3); and plumbing lines (Zone 4).

To enable science operations, the WRP (Zone 3) must first be heated to +25°C before the rotors can begin to spin; this heating step initiates both GCMS and LDMS experiments. During GCMS operations, Zones 1 and 4 are also activated to achieve ≥135°C along the sample path, extending from the GC column to the ion trap analyzer. In contrast, during LDMS operations, no hardware is heated actively other than the WRP. In between science experiments, Zone 2 may be engaged opportunistically for clean-up operations to bake out accumulated volatile contamination within the sensor. The temperatures of optical components within the LH are managed by a separate service; thermal manipulation of this cavity serves to control the output energy of the laser.

#### 6.4.4. Driving electronics

The driving and control electronics for MOMA are generally broken up into two electronic boxes to allow optimal accommodation in the ExoMars rover and to provide efficient delivery of relevant signals to the sensor. These electronic boxes were built and developed through collaboration between GSFC, Space Physics Research Laboratory at the University of Michigan, and Battel Engineering.

The MEB houses the primary power supply (PS), command and data handling board (CDH), motor and analog interface (MAIF), and LPU. The PS takes in the bus power from the rover and distributes it to various components of the instrument. The CDH houses the main computer that controls all of the MOMA subsystem operations and sends the acquired data and telemetry back to the rover. The MAIF controls the WRP, the various valves that are part of the MS GC and LDI (Laser Desorption and Ionization) inlet, the micropirani pressure gauge, and the heaters that are part of the MS and its GC inlet. Finally, the LPU provides the diode pump pulse and control and monitoring of the physical LH that is mounted to the MS housing.

The secondary electronic box (SEB) houses the control board (CTL), power and high-voltage (PH) board, and filament bias (FB) board. The CTL contains all of the low-level MS control electronics that handle the sequencing, monitoring, and signal counting necessary to perform the ion trap MS experiment. The PH and FB boards together provide all of the lens voltages that are necessary to generate, trap, and detect ions from the MS.

There are a few other smaller distributed pieces of electronics necessary to complete the operation of MOMA. Specifically, the GC contains its own set of control electronics that interface to the CDH to receive instruction and provide data and telemetry back to the central control computer. There is also a separate RF (Radio Frequency) PS to provide RF voltages to the rods necessary to trap and eject ions in the ion trap. The RF supply is physically mounted to the MS housing to minimize power losses and optimize efficiency. Finally, there is a dedicated set of detection electronics with two duplicate circuits (one for each detection channel) that amplify and discriminate the pulses generated by the CEMs and feed counters in the CTL. The detection electronics are also physically mounted to the MS housing to reduce the signal-out cable length and minimize noise and signal loss in these very low-level signals.

## 7. Operation of MOMA on the Surface of Mars

### 7.1. Expected measurement scenarios

The ExoMars rover will physically and chemically characterize the martian terrain encountered during a nominal 218 sol Reference Surface Mission (RSM). The RSM comprises two types of scientific and engineering sequences: experiment cycles and vertical surveys. During an experiment cycle, anticipated to last 13–18 sols, the rover will (1) traverse to a preidentified location of interest based on orbital and rover data, (2) perform a full measurement cycle, and (3) transmit all of the scientific, housekeeping, and navigation data to Earth. Within a measurement cycle, crushed powder is produced from a drill sample and delivered to the refillable sample tray (a.k.a. refillable container) inside the UCZ. Before sample analysis by MOMA, the MicrOmega and RLS instruments will conduct contextual measurements of mineralogy and organic functional groups that may be present (Vago *et al.*, 2016). In cases where uplink and downlink schedules permit a tactical response during the science operations cycle, quicklook snapshots of these data may be used to select or tune the MOMA analytical approach.

Following special, high-priority experiment cycles, the mission may elect to perform a vertical survey to obtain deep samples. A minimum of two vertical surveys are allocated in the RSM. The purpose of the vertical survey is to fully characterize the martian soil's geochemical and organic distribution as a function of depth, particularly to characterize the ratio between organic and oxidant molecules. This vertical survey obtains crushed regolith samples from depths of 0 (*i.e.*, surface-bounded), 50, 100, 150, and 200 cm and delivers these samples to each of the analytical laboratory instruments. MOMA will provide information on organic content through LDMS and GCMS modes described below.

### 7.2. Sequenced operational modes

**FIG. 10.**
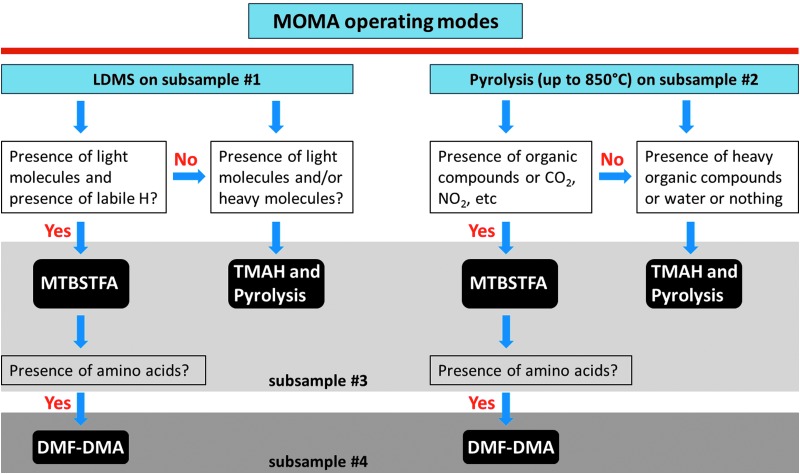
Flow diagram for MOMA operating modes. Quick survey analyses will only use subsample #1. Experiment cycles and vertical surveys will use subsamples #1 and #2 and, depending on quick assessment of the data, also subsamples #3 and #4. Direct detection of CO_2_ and NO_2_ (mentioned in the flow diagram) may or may not be possible, depending on the mass cutoff of the MS.

The MOMA instrument can perform complementary experiments, independently or sequentially, to analyze the martian soil (Fig. 10). The two modes are LDMS and GCMS. GCMS offers two types of sample analyses (pyrolysis and wet chemistry) that allow MOMA to target specific molecules such as volatile compounds, polar compounds, or macromolecules. MOMA examines solid crushed sample exclusively; it does not include atmospheric analyses. LDMS desorbs and analyzes only the outer surface (μm scales) of sample grains. In GCMS mode, the ovens (pyrolysis or wet chemistry) receive ∼100 mm^3^ of loosely packed sample. By design, MOMA's analytical capabilities are not applied to the very same sample, but only to different aliquots of a given crushed drill core. The extent of MOMA analysis of a sample depends on tactical science decisions in concert with the mission team. Nominally, LDMS is the first MOMA mode to be applied to a given sample (referred to as subsample #1). This will give us information about the sample's potential organic content. In particular, LDMS targets heavier nonvolatile compounds that may be present in the martian sediment. LDMS additionally provides information about the presence or absence of labile hydrogen and polar compounds. LDMS results will thus drive the decision of performing GCMS analysis.

If pyrolysis GCMS is to be performed (subsample #2), several GC parameters have to be chosen, including column and trap type, temperature ramp profile, and carrier gas pressure. The column and trap choices will depend on the kind of molecules present (low-mass volatile, chiral, or refractory compounds). These options will depend, in part, on initial results from the LDMS analysis.

If labile and/or polar compounds are found in the subsample, then an additional subsample (#3) may be analyzed by using a derivatization or thermochemolysis reagent. Such a decision would be discovery driven and made in the context of all mission science data and operational reviews. A sample sufficiently rich in bound water (*e.g.*, clay minerals) could inhibit the MTBSTFA or DMF-DMA reaction if release temperatures were comparable. In this case, TMAH, which is relatively immune to water, could be chosen. Otherwise, MTBSTFA would be used. If the MTBSTFA reaction leads to detection of amino acids in the sample, an additional subsample (#4) could be used with the DMF-DMA reagent to allow a chiral separation in conjunction with the enantioselective GC column (CP Chirasil-Dex CB).

If heavier compounds such as macromolecules are detected, then thermochemolysis and pyrolysis would likely be used to reach the most refractory compounds of the sample (Fig. 10).

### 7.3. Synergy with other instruments

The samples most likely to contain undegraded organic molecules, as they have been protected from cosmic and UV radiation, will be deep drill samples. Therefore, the decision to drill as taken from WISDOM radar data (Water Ice Subsurface Deposit Observation on Mars; Vago *et al.*, 2017) will affect overall operational decisions made by MOMA. The most extensive MOMA investigations will probably be performed on these samples.

The presence of water and hydrated minerals as detected by the various instruments on the rover (Vago *et al.*, 2017), ISEM (infrared spectrometer for ExoMars), Ma_MISS (Mars Multispectral Imager for Subsurface Studies), MicrOmega, ADRON (Active Detector for gamma Rays and Neutrons), and RLS, will affect the experimental strategy in different ways. Most surface samples analyzed to date do contain oxychlorine species (including perchlorates and chlorates) at various abundance levels (probably ranging from almost zero to a few wt %). If the NIR reflectance as measured by MicrOmega is indeed consistent with concentrated perchlorate (Morris *et al.*, 2009; Cull *et al.*, 2010), then only LDMS experiments may be performed, possibly followed by low-temperature GCMS experiments. However, if no traces of hydrated oxychlorines can be detected and if most water is apparently held by other hydrated compounds (such as smectites or sulfates), default MOMA experiments will be conducted. In any case, excess water from the sample is generally unsafe for the instrument; as such, preheating of the sample will be necessary, with the disadvantage of some volatile organic molecules potentially being lost. Preheating to remove water might also be necessary when operating MOMA in the derivatization mode (MTBSTFA, DMF-DMA) if the water is not removed during the default temperature rise to the melting point of the capsule.

#### 7.3.1. Data interpretation

Information on minerals as provided by the above listed instruments will affect the interpretation of LDMS data. Raw LDMS data comprise a mix of peaks originating from both organic and inorganic molecules. Any information on the sample's mineral content will help more completely identify which peaks in the spectra originate from minerals and zero in on organic signals. Spatially precise mineralogical data obtained by MicrOmega and RLS will support MOMA LDMS point-by-point analyses (typically 5–10 adjacent RLS spots and 10–20 MicrOmega pixels will span the ellipse probed by MOMA LDMS). In addition to any inorganic signal, MicrOmega and RLS are expected to provide information on organic molecules.

In case of the detection of indigenous martian organic molecules by MOMA, the overall geological context such as the depositional environment, including mineralogy from outcrop to grain scale, will be of great importance (Siljeström *et al.*, 2014). This will provide further constraints on the abiotic versus biological origin of these molecules. Although some specific organic molecules might act as biosignatures on their own, for example, amino acids in ee or hopanes, many organic molecules, for example, PAHs or macromolecular carbon (known to occur on Mars and in martian meteorites, Steele *et al.*, 2012; Freissinet *et al.*, 2015), might be part of meteoritic infall or may have formed by abiotic processes on Mars or even by diagenetic breakdown of biological molecules. Therefore, any information provided by other instruments on the rover will be key to the interpretation of the origin of the organic matter. For example, PanCam (panoramic camera system) together with ISEM, ADRON, and CLUPI (CLose-Up Imager) will provide the overall geological context of the sample location on Mars (Vago *et al.*, 2017), and although MOMA might provide some information regarding mineralogy, other instruments on the rover are better equipped to do so, such as ISEM on the outcrop scale, Ma_MISS from the drill hole, and MicrOmega and RLS on the single-grain scale. All three ALD instruments will probe the same delivered subsample, providing *in situ* correlation between data from these instruments.

Even in the case of detection of a clear biosignature, such as a hopane, the direct connection of this signature with a morphological feature as spotted by either CLUPI or MicrOmega will strengthen the case for biology. In addition to the mineralogical context, RLS will also help MOMA in detection and characterization of any organic matter in samples (Edwards *et al.*, 2012, 2013). In particular, it can help assess crystallinity and thermal maturity of any macromolecular organic matter. Any information on the organic content from MOMA will be important to decide if the rover should stay at the current location or move on to another area.

### 7.4. LDMS operations on Mars and test experiments on LDMS prototype system

MOMA's LDMS mode (Li *et al.*, 2017) provides a rapid and essentially nondestructive method to analyze the higher-molecular-weight (up to 1000 u) organic content of a crushed sample before detailed analysis by pyrolysis GCMS. The LDMS experiment consists of the following major steps: (1) LDMS analysis of the MOMA calibration target at a fixed laser energy, (2) rotation of the carousel to a designated sample location in the refillable container, (3) LDMS analysis under an autonomous ramp of increasing laser shot count and pulse energy, and (4) repetition of (2) and (3) for at least two addition sample locations. Alternatively, the number of sample locations could be increased to 10, providing more coverage and/or finer analysis at the cost of fewer spectra obtained per location. The autonomous ramp is a type of automatic gain control (AGC) for LDMS. Laser desorption/ionization typically exhibits threshold behavior: signal levels increase exponentially immediately above a sample-dependent threshold laser fluence. Sample factors that impact the threshold fluence include chemical composition (matrix), morphology, and microstructure, with mixed-phase samples exhibiting more diffuse threshold behavior than simple and homogeneous samples. Optimal performance of MOMA when ion signals exceed the background sufficiently for detection, but do not overfill the trap capacity, is realized slightly above the LDI threshold. The laser AGC algorithm includes a data-dependent feedback loop that adjusts the laser energy as well as the number of pulses per spectrum based on the appropriate ion count. No human intervention or operational delay is needed.

LDMS data are acquired autonomously during a sample run comprising several hundred individual mass spectra produced from a few thousand laser pulses. After data collected during the most recent sol are downlinked to the ExoMars science operations center, the MOMA science team will have an opportunity to provide a preliminary processing step to generate quicklook products suitable for rapid decision-making. Aside from basic health and quality checks, LDMS data will be reviewed for general features of interest, and a consensus decision will be made to recommend one of three potential next steps: (1) pursue a detailed follow-up LDMS investigation of a set of individual peaks or mass-to-charge ranges of interest by SWIFT isolation and MS/MS fragment analysis (Li *et al.*, 2017), (2) investigate the organic composition further by delivering sample from the crusher to a pyrolysis oven for the MOMA GC mode, or (3) defer or end the investigation of the current sample in favor of further analyses by other ALD instruments and/or continued rover operations in pursuit of a new sample. Option (1) would likely be invoked for a rich mass spectrum that exhibited one or several high-intensity peaks at mid- or high-molecular-weight matching patterns consistent with organic composition. Option (2) would most likely be implemented as a subsequent experiment following SWIFT and MS/MS or for a sample that exhibited a broadly rich sequence of peaks across the LDI mass range. Option (3) would most likely be pursued in the case that few features were observed in LDI mode and it was deemed in the mission's interests to conserve consumable MOMA resources, such as ovens or carrier gas. In the case where the Raman or MicrOmega spectrometers subsequently identified additional points of interest on the sample that were also within the MOMA laser field of view, Option (3) could also result in a return to LDMS runs on the same sample.

During development of the relatively new application of Mars ambient LDI of mineralogic samples using the MOMA LIT, a library of test data acquired by using natural and synthetic analog samples has been started. This process is based upon empirical comparisons of spectra of mineral matrices with and without organic spikes, built up from simple combinations using pure phases. Previous work has demonstrated that LDI enables detection of trace amounts (ppmw levels) of complex organics at fine scales in mineral matrices, even in the presence of 1 wt % of perchlorate salt (Li *et al.*, 2015).

**FIG. 11.**
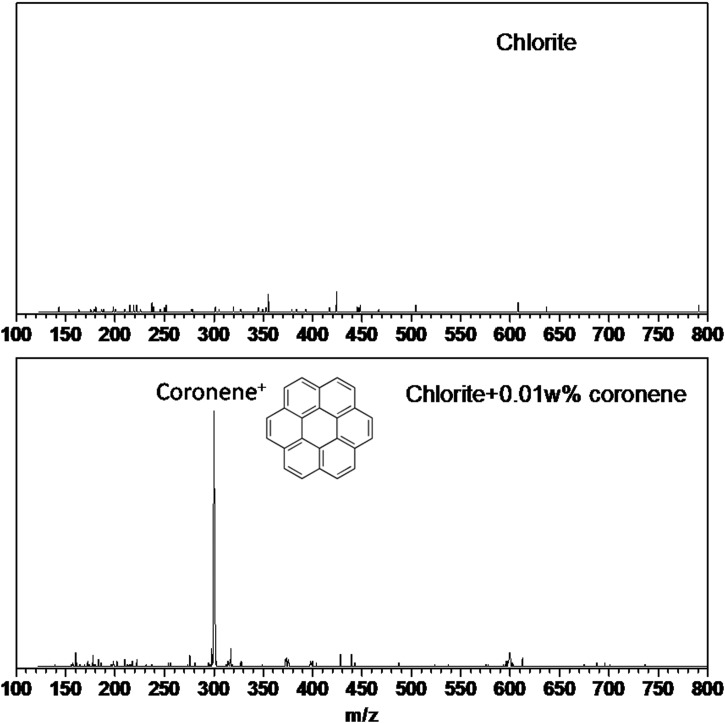
Positive ion LDMS spectrum of chlorite alone (top) and chlorite doped 0.01 w% with example PAH coronene. In this example case, the Coronene molecular ion is readily detectable above the very low background contributed by the mineral analog. LDMS, laser desorption mass spectrometry.

Figure 11 shows LDI mass spectra for another example: a chlorite mineral spiked with 0.01 wt % coronene. Chlorite clays are of high interest for potential detection on Mars and in particular could be a pointer to burial, and organic preservation, processes if found in a bedding relationship with smectite clays, already identified at Oxia Planum (Section 1). Coronene is readily detected at this concentration level at low-to-moderate laser pulse energies relative to the MOMA laser range (see also Li *et al.*, 2017). The mass spectrum of the spiked sample showed a dominant base peak associated with the molecular ion of coronene at *m/z* 300. Data from the pure chlorite powder under the same laser conditions confirm that the mass signature at *m/z* 300 arises solely from organic parent ions without significant interference.

Other types of mineral phases can yield a host of inorganic molecular ions, primarily cation oxide clusters, under moderate fluence laser desorption, for example, Fe_x_ and Fe_x_O_y_ from magnetite or Mg_x_O_y_ and Mg_x_Si_y_O_z_ from forsterite with x, y, and z being small integers (Goetz *et al.*, 2016a, 2017). These clusters serve to probe the sample's mineral composition and can be used to identify minor phases as well as provide a localized association with any detected organics. When a putative organic compound is identified in such an LDMS matrix background, techniques such as SWIFT and MS/MS may be employed to isolate it to minimize any potential interference and resolve the structure of the organic species. With the recent downselection of candidate landing sites for the rover (Section 1), the MOMA team is focusing testing efforts on appropriate analogs of the materials identified from orbit. Through measurement of the mineral matrix- and laser-induced effects on organic sensitivities, procedures for systematically reducing data from these more complex real-world samples are being established.

### 7.5. Test experiments on GCMS prototype system: a case study

Here we present a case study to illustrate MOMA GCMS capabilities on a specific type of sedimentary rock. The goal of this study was threefold: first, to demonstrate how the analytical methods work independently; second, to compare different MOMA GCMS methods (pyrolysis and derivatization) by using only one sample; and last, to learn about advantages and disadvantages of these methods. The study was carried out in a laboratory configuration that mimics the MOMA flight instrument and was designed to gain qualitative information only.

#### 7.5.1. Sample and setup

The case study was carried out on a silica-dominated black radiolarian chert from the Holy Cross Mountains in central Poland near the village of Zalesie Nowe (for details, see Kremer, 2005; Kremer and Kazmierczak, 2005; and Bauersachs *et al.*, 2009). The sample has a total organic carbon content of 0.49% (determined by thermal decomposition and combustion; Activation Laboratories Ltd., Canada). Despite its biogenic origin, the sample can serve as a Mars analog sample as the original biogenic chert has been completely recrystallized since its deposition and diagenesis in Silurian time. Opaline silica deposits are known to exist on Mars and have been detected both from orbit (Milliken *et al.*, 2008) and *in situ* by the Mars Exploration Rover Spirit (Squyres *et al.*, 2008). The relatively high total organic carbon content (in comparison with Mars) is beneficial for analysis under common laboratory conditions to reach a better signal/noise or sample/contamination ratio. Before the analyses, the sample was manually powdered (<50 μm) with a mortar and pestle that were precleaned with isopropanol to prevent any contamination by organics.

The measurements were performed with a flight analog system (FAS; only MOMA pyrolysis/derivatization oven, adsorption trap, and TS) connected to a Varian CP-3800 GC coupled to a Varian 240-MS/4000 MS. The GC column was a Varian Factor Four™ VF-5ms (30 m in length, inner diameter 0.25 mm, 0.25 μm film thickness) that is similar to the MOMA Restek MXT 5 column (Table 3). Helium was used as the carrier gas. The gas flow could be switched between internal (GCMS only) and external (FAS and GCMS). The FAS consists of a flight-like reusable MOMA oven and MOMA TS, including the adsorption trap (filled with Tenax GR adsorbent; see also Section 6.2). Closing and reopening the oven were possible by hand with a flight-like zirconium ball sealing. The trap was cooled down by a Peltier element and heated to ∼150°C by an external heating element (heating rate 200°C/min). The major differences between the FAS and the flight system are (1) the wet chemistry reagent is already added to the sample at room temperature (instead of being released by the MOMA flight capsule at temperatures above ∼140°C, Section 6.1.3 and Fig. 4), (2) the volatiles need to pass through the whole trap to get into the GC (instead of being subjected to flow inversion that is referred to as backflush, Section 6.2.2.2), (3) the trap can only be heated to ∼150°C (instead of 300°C, as planned during Mars surface operations), and (4) the capillary transfer lines from oven to trap and trap to GC in the FAS can only be heated to 110°C (instead of ≥135°C when operated on Mars).

#### 7.5.2. Methods

##### 7.5.2.1. Stepwise pyrolysis

Three measurement cycles were performed on the same sample material with successive pyrolysis temperatures (300°C/500°C/700°C), without opening the system in between. A pyrolysis step at 300°C is useful for removing volatile components and any contaminants from the sample (*e.g.*, Brocks *et al.*, 2003). The oven was filled with 3 to 3.5 mg of the sample and sealed. The oven could have received ∼20 times more sample (Section 6.1). Smaller sample masses compensated somewhat for the high abundance (∼5000 ppm C_tot_) of carbonaceous material in the sample. External helium flow was then activated and the trap was cooled down. When the trap reached 0°C, pyrolysis of the sample with a heating rate of 300°C/min was activated and held at the relevant temperature for 10 s. Then, flash heating of the trap was synchronized with the start of GCMS data acquisition. After 45 s into the GC run, the helium flow was switched to internal to guarantee optimal helium flow for the entire measurement.

##### 7.5.2.2. Derivatization/thermochemolysis

The following wet chemistry reagents were used:

• MTBSTFA (>97%, Sigma-Aldrich) with DMF (>99.5%, Thermo Scientific™) in a 3:1 (V:V) mixture
• DMF-DMA (Sigma-Aldrich)
• TMAH (25 wt % in methanol, Sigma-Aldrich)

Three to 3.5 mg of sample was filled into the oven and predried at 80°C for 10 min with the oven disconnected from the TS; 3.5 μL of liquid chemical reagent was added to the sample and the oven was sealed. Hence, we used the same amount of sample as for the pyrolysis experiments (Section 7.5.2.1) and about five times less reagent than released by the MOMA flight capsules (∼15 μL, Section 6.1.3). These experimental parameters follow expected conditions during MOMA surface operations as closely as possible given (i) the real differences between FAS and MOMA flight system (Section 7.5.1), (ii) the differences between our test sample (Silurian radiolarite) and expected martian subsurface samples, and (iii) practical constraints in the laboratory (less than ∼3 μL could not be handled in a reproducible way, even with microliter syringes). External helium flow was activated, the trap was cooled down to 0°C, and derivatization was initiated by heating the oven/sample to the desired temperature. The following reaction times and temperatures were chosen for these experiments: 10 min at 250°C for MTBSTFA/DMF; 4 min at 140°C for DMF-DMA; 40 s at 600°C for TMAH with a general heating rate of 300°C/min for the oven, again following expected MOMA protocols (Sections 6.1.1 and 6.1.2). After the reaction, flash heating of the trap and GCMS data acquisition started simultaneously. After 1 min into the GC run, the helium flow was switched back to internal.

##### 7.5.2.3. GCMS parameters

The carrier gas flow rate was set to 2 mL/min (for all measurements) with a split ratio of 30; the injector temperature was 250°C; and the GC column was heated from 30°C to 250°C with a 10°C/min ramp, holding at the maximum temperature for 5 min. Starting points for ionization (MS filament switched on) were 1 min for pyrolysis, 8 min for MTBSTFA/DMF and DMF-DMA, and 7 min for TMAH (solvent delay). Full-scan mass spectra were recorded with ionization fast-scan mode and a scan time of 0.58 s over a mass range of *m/z* 35 to 1000.

Before each analysis, a cleaning run and a system blank run (GCMS and FAS) were performed to check for contamination. Additional blank runs were performed for every derivatization analysis (GCMS, FAS with wet chemistry reagents).

#### 7.5.3. Results and discussion

##### 7.5.3.1. Pyrolysis

**FIG. 12.**
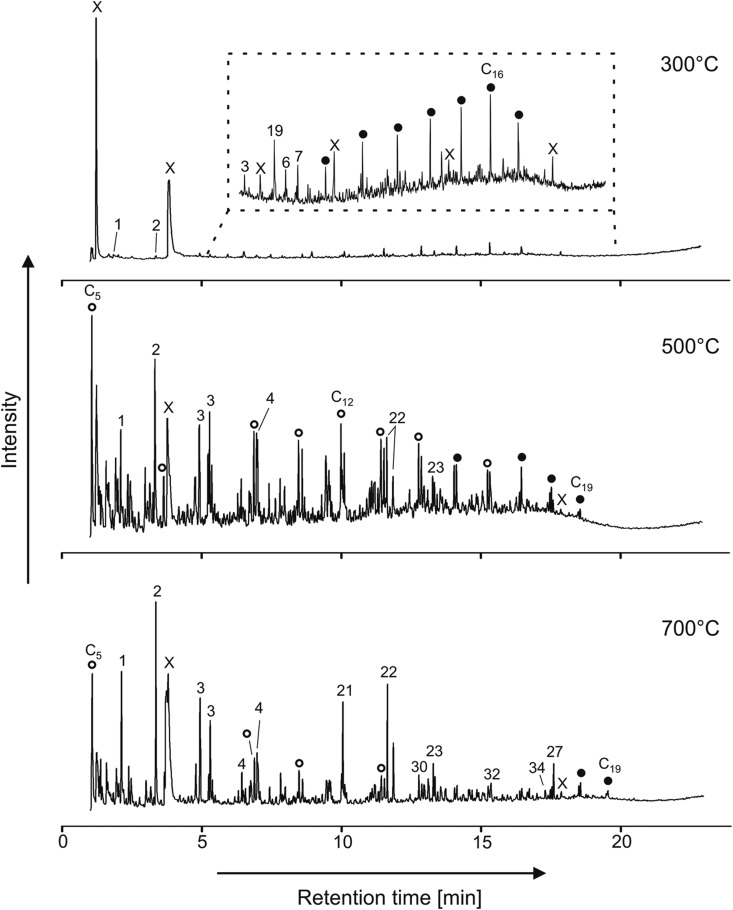
GCMS chromatograms (total ion current) of stepwise pyrolysis (300°C, 500°C, 700°C). Open and filled circles indicate *n*-alkenes and n-alkanes, respectively; X indicates contaminants; and numbers indicate aromatic compounds (Table 6). Note that in the 500°C and 700°C chromatograms, only a small selection of peaks are marked; for further details, see Figure 13.

The stepwise pyrolysis displayed a huge inventory of aliphatic and aromatic hydrocarbons. A selection of the most abundant and important ones are presented in Figure 12. Additionally, a significant amount of contamination signals were identified. Especially in the 300°C step, abundant acetone and phthalate peaks were present that are probably contamination from sample collection and preparation (*i.e.*, plastic boxes, tool cleaning; Fig. 12). Other contamination (mostly siloxanes and DMF) is visible in each heating step. Siloxane is a typical signal from column degradation, whereas the DMF signal is caused by a carryover effect from the trap and derives from prior measurements with MTBSTFA/DMF.

**FIG. 13.**
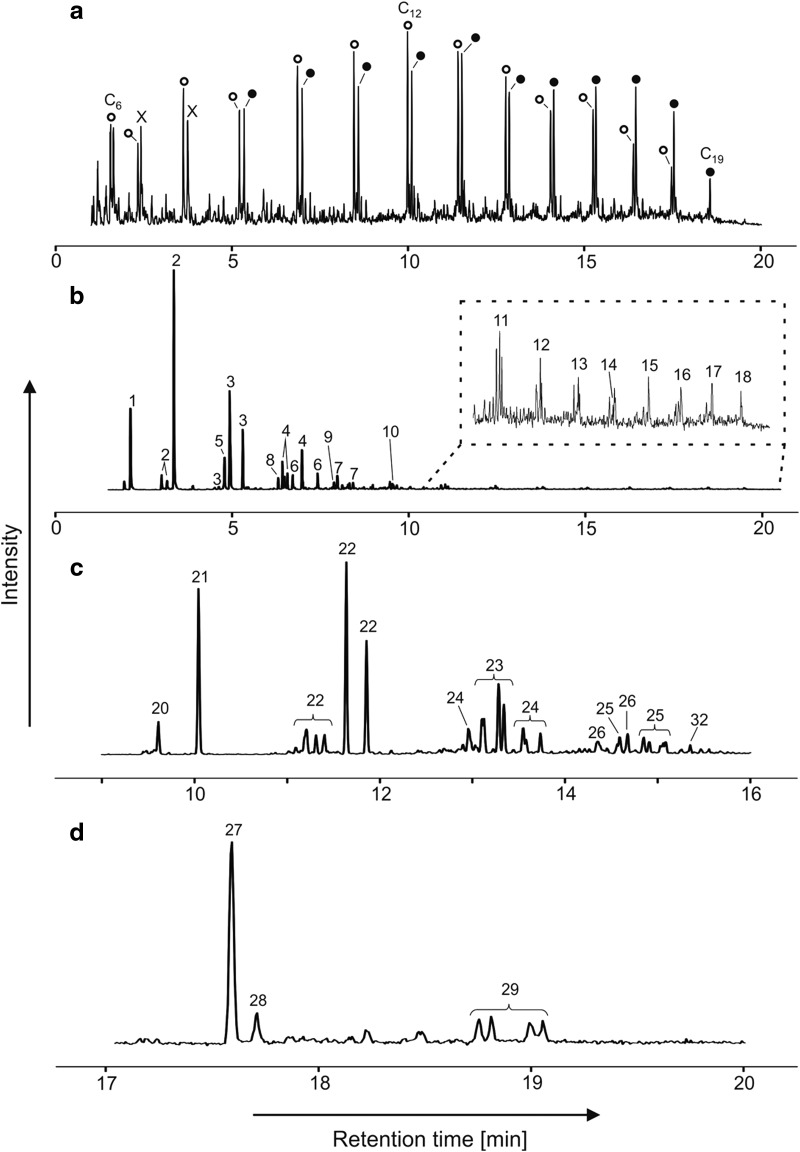
GCMS chromatograms of stepwise pyrolysis filtered for certain compounds. **(a)** *n*-Alkenes and *n*-alkanes (500°C, *m/z* 69, 71, 83, 85), **(b)** benzene and alkylbenzenes (700°C, *m/z* 78, 91, 92, 105, 106, 119, 120), **(c)** naphthalene and alkylnaphthalenes (700°C, *m/z* 128, 141, 142, 155, 156, 170), and **(d)** phenanthrene, anthracene, and methylphenanthrenes (700°C, *m/z* 178, 191, 192). Open and filled circles indicate *n*-alkenes and *n*-alkanes, respectively; X indicates contaminants; numbers indicate aromatic compounds (Table 6).

Aliphatic hydrocarbons from the sample are mainly represented by *n*-alkanes/*n*-alkenes (Figs. 12 and 13a). It should be noted that the *n*-alkenes in this case are by-products of cracking hydrocarbon compounds (*e.g.*, *n*-alkanes) from kerogen (*i.e.*, refractory material not extractable by organic solvent [Durand, 1980]) by open-system pyrolysis and are therefore not indicative of the original hydrocarbon inventory of the sample (*e.g.*, Burnham *et al.*, 1982; Huizinga *et al.*, 1988). This bias is intrinsic to the pyrolysis technique. Whereas the 300°C step only shows *n*-alkanes in the range of C_11_ to C_17_ with low abundance (Fig. 12, 300°C), higher-temperature pyrolysis yields much higher abundance levels of *n*-alkane/*n*-alkene doublets in the range between C_5_ and C_19_. Particularly, the 500°C pyrolysis run is dominated by *n*-alkanes/*n*-alkenes with a predominance of *n*-alkenes in the range of C_10_ to C_14_ (Figs. 12 and 13a). *n*-Alkanes are not detectable in at least the first 5 min of each pyrolysis run.

The abundance of aromatic hydrocarbons released from the sample increases with increasing temperature of the single steps. Only traces of benzene (1) and toluene (2) are found in the 300°C step, whereas aromatic hydrocarbons are the dominant compounds in the 700°C run (Fig. 12). The predominance of aromatic compounds at 700°C is probably (at least partly) an artifact from pyrolysis due to cyclization and aromatization of unsaturated hydrocarbons during heating (*e.g.*, Sáiz-Jiménez, 1994; Hartgers *et al.*, 1994, 1995). In particular, very high amounts of toluene, benzene, and methylnaphthalene could be seen (Fig. 12, 700°C). Besides these compounds, a variety of benzene/alkylbenzenes (Fig. 13b), naphthalene/alkylnaphthalenes (Fig. 13c), and phenanthrene/methylphenanthrenes/anthracene (Fig. 13d) were observed. For an overview of the most abundant aromatic hydrocarbons detected, see Table 6.

**Table 6.** Peak Labels and Compound Names of Aromatic Hydrocarbons

*Peak label*	*Compound*	*Peak label*	*Compound*
1	Benzene	18	Tridecylbenzene
2	Toluene	19	Benzaldehyde
3	Xylene	20	Naphthalene
4	Trimethylbenzene	21	Naphthalene
5	Ethylbenzene	22	Methylnaphthalene
6	Ethyl-methylbenzene	23	Dimethylnaphthalene
7	methyl-(1-methylethyl)benzene	24	Ethylnaphthalene
8	Propylbenzene	25	Trimethylnaphthalene
9	Butylbenzene	26	(1-Methylethyl)-naphthalene
10	Pentylbenzene	27	Phenanthrene
11	Hexylbenzene	28	Anthracene
12	Heptylbenzene	29	Methylphenanthrene
13	Octylbenzene	30	Biphenyl
14	Nonylbenzene	31	Dibenzofuran
15	Decylbenzene	32	Fluorene
16	Undecylbenzene	33	Methyldibenzofuran
17	Dodecylbenzene	34	Dibenzothiophene

Two peaks (20 and 21) have been labeled confidently as naphthalene, although the appearance of two peaks for this compound is poorly understood.

The results found from the FAS pyrolysis experiments are comparable with data from analysis performed on a similar type of sample (mudstones and black shales from the Ordovician–Silurian boundary, Holy Cross Mountains, Poland) with standard pyrolysis instruments (Mustafa *et al.*, 2015). The authors found a similar inventory of aliphatic and aromatic hydrocarbon compounds. Few differences could be seen in the range of organic compounds (*e.g.*, *n*-alkanes, alkylbenzenes) detected where the FAS analysis showed distribution over a wider range of chain lengths. Overall, stepwise pyrolysis experiments have confirmed that this method is capable of giving clear insight into and a good overview of the hydrocarbon inventory of an unknown sample. However, a distinction whether hydrocarbons (or possible biomarkers) derive from the bitumen or kerogen fraction cannot be done. For this purpose, a pre-extraction of samples would be necessary to remove the bitumen fraction (*i.e.*, solvent-extractable organics [Durand, 1980]), which of course is not possible for MOMA. Nonetheless, it would be useful to investigate the kerogen separately as it is assumed to be more resistant against maturation, to be immobile, and therefore syngenetic to the host rock and less affected by contamination compared with the bitumen (Brocks *et al.*, 2003; Love *et al.*, 2005; Marshall *et al.*, 2007). These important points clearly highlight an inherent weakness of the MOMA instrument that is like any other remotely operated space instrument, a trade-off between low complexity and science return.

It can only be speculated here that compounds (including alkanes and aromatics) released in the 300°C run were contamination or probably released from the free bitumen fraction (not chemically bound) and therefore less thermal energy is needed for release. Additional compounds released at 500°C can possibly be organics from the bitumen fraction (also with higher molecular weight) or derive from the kerogen and/or mineral-bound bitumen, whereas the compounds found in the 700°C run may only derive from kerogen.

##### 7.5.3.2. Derivatization and thermochemolysis

**FIG. 14.**
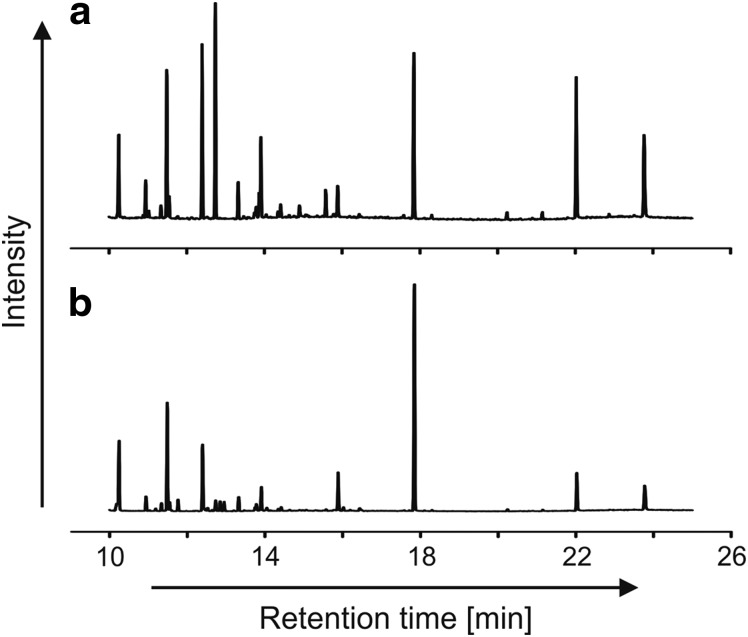
GCMS chromatograms (total ion current) of the MTBSTFA/DMF derivatization. **(a)** Blank run with MTBSTFA/DMF, **(b)** derivatization of the sample with MTBSTFA/DMF. No major differences are noted.

The analysis with MTBSTFA/DMF derivatization does not show any usable results. Only a low abundance of sulfuric acid, few fatty acids, and alcohols, which are most likely contamination from sample processing, could be detected. Most peaks were also present in the blanks (Fig. 14a, b). The same goes for the analysis with DMF-DMA. A possible explanation for this negative result is that the sample did not contain compounds detectable with derivatization (no compounds with functional groups) as the sample has been too weathered and diagenetically altered to contain any compounds with functional groups. Alternatively, the low temperature of the derivatization procedure (250°C for MTBSTFA/DMF, 140°C for DMF-DMA) did not allow the molecules to be extracted from the sample and thus functional groups of the molecules were not available to the derivatization reagent. An additional step of preheating the sample to higher temperature may be necessary to thermodesorb the molecules before derivatization. Further investigations are needed to optimize the method. However, some fatty acids (most likely contaminants) were identified, which confirms the efficiency of the derivatization method.

The case study showed that some issues persist and need to be considered before *in situ* derivatization. The samples need to be dried out carefully (not too much blowoff from the sample) before derivatization to avoid total consumption of the reagent by water. Additionally, carryover effects need to be considered when using a surplus of derivatization reagents (Fig. 14a) as this will also derivatize residual material from prior analysis out of the trap, the GC column, and all low-temperature areas (*e.g.*, transfer lines), leading to cross-contamination of samples.

**FIG. 15.**
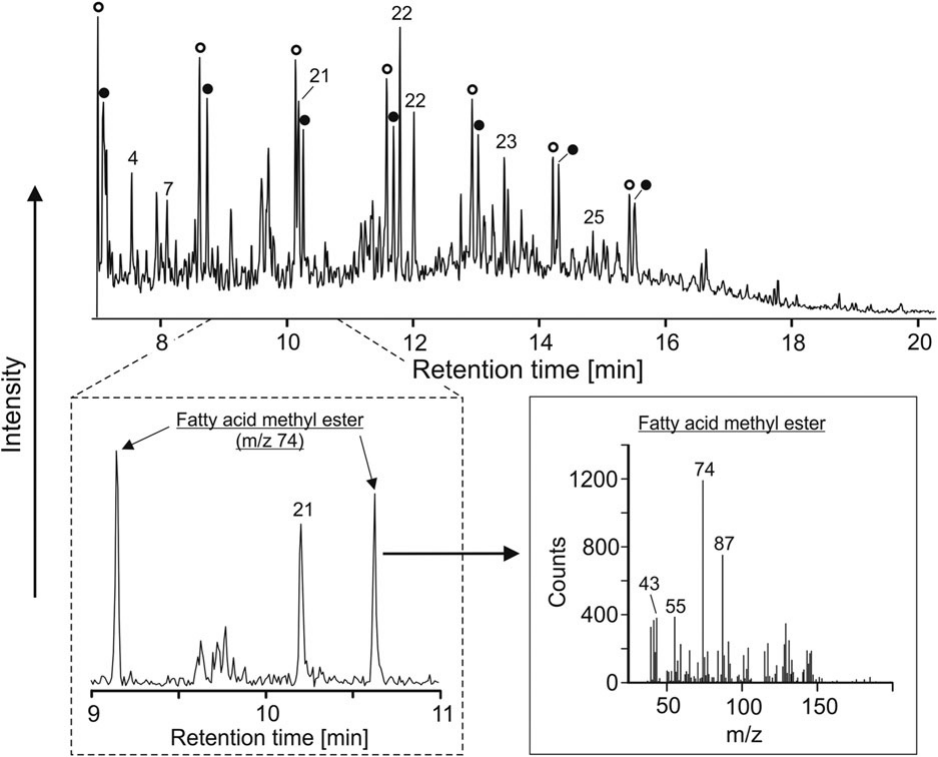
GCMS chromatograms of TMAH thermochemolysis. Top: total ion current, similar to pyrolysis. Bottom left: part of the chromatogram (*m/z* 74) with identified fatty acid methyl esters. Bottom right: corresponding mass spectrum of fatty acid methyl esters. Open and filled circles indicate *n*-alkenes and *n*-alkanes, respectively; numbers indicate aromatic compounds (Table 6). TMAH, tetramethylammonium hydroxide.

Thermochemolysis of samples analyzed with TMAH showed mostly the same compounds as the stepwise pyrolysis (Fig. 15, see also Section 7.5.3.1). However, one crucial difference is that two fatty acid methyl esters could be identified (Fig. 15), but they only show low abundance. An exact classification of these was not possible because of co-eluation with aromatic compounds. It is likely that these two fatty acids are contamination from sample preparation as the sample is otherwise free from functionalized compounds. Nonetheless, we were able to analyze and identify these fatty acids by *in situ* thermochemolysis.

## 8. Discussion

MOMA is a mass spectrometer-based instrument onboard the ExoMars rover designed for *in situ* analysis of martian samples. This article describes the scope and development of MOMA from its science requirements to the complete instrument as currently expected (about 1 year before completion of the integrated MOMA FM). We have described MOMA's major subsystems (ovens, TS, GC, laser, and MS) and test experiments conducted on subsystem test modules, as well as coupling campaigns between selected subsystem modules. However, tests of the fully integrated instrument are yet to come. The overarching goal of MOMA is to characterize the inventory of organic material in martian sediments. According to the ExoMars mission goals (Vago *et al.*, 2017), such characterization shall be detailed enough to address and constrain the sources (biotic or abiotic) of that material and, in particular, to address the question of whether or not that material is formed from biologic activity. The task is complex as up to now, only little organic material (∼150 ppbw of chlorobenzene and tens of ppbw of C_2_–C_4_ chlorohydrocarbons) has been identified *in situ* in martian sediments that has clearly been recognized to belong to the indigenous organic part of these sediments (Freissinet *et al.*, 2015). The organic molecules detected so far have been thermally released from its sedimentary environment. We still do not know if these molecules occur as discrete molecules in the rock or if they are part of a macromolecular organic compound, although the latter seems to be more likely. Hence, we do not know the nature of the organic material contained in the martian sediment. The methods applied to date (especially pyrolysis-GCMS onboard the Viking landers and the Curiosity Rover) are fairly destructive, and consequently important information on the potential sources of this organic material is lost. In this respect, MOMA will represent a clear advance as it combines destructive methods such as classical pyrolysis (Viking landers, MSL-SAM) and pyrolysis–derivatization (MSL-SAM) with a less destructive method, that is, LDMS, which allows laser-ablated, fairly large, and intact molecular fragments to be detected and characterized by the MS. Tandem mass spectrometry can then be used to further characterize these molecules. This method has never been applied to martian material by any previous landed mission.

Classical pyrolysis-GCMS-based techniques have two major drawbacks: (1) only volatile molecules can be detected and, moreover, only nonpolar molecules can get through the GC columns to the detector and (2) heating of samples to temperatures beyond ∼200–300°C causes oxidative destruction (chlorination and combustion) of organic compounds by (per)chlorates. These salts have been detected (directly and indirectly) by previous missions (especially Phoenix and Curiosity) and are believed to occur at all latitudes and in sediments of any age (Glavin *et al.*, 2013; Archer *et al.*, 2016).

The LDMS technique is not affected by these drawbacks as this technique is insensitive to the presence of (per)chlorates (Li *et al.*, 2015) and to the polarity of laser-desorbed molecules. Additionally, MOMA tackles the above drawbacks by offering a broader set of derivatization agents than currently available on SAM: (ad 1) different derivatization agents target different types of molecules, including molecules of different polarity; and (ad 2) MOMA offers two derivatization techniques that both occur at rather low temperature: MTBSTFA/DMF (∼250°C) and DMF-DMA (∼140°C). Based on SAM data (acquired on 10 samples, Archer *et al.*, 2016), both temperatures are below the temperature where most samples start to decompose their (per)chlorates and release O_2_ that leads to combustion of organic molecules.

In addition to the mass range and polarity of any martian organic compounds, we do not know if the organic material detected so far represents a homogeneously distributed discrete phase (such as a mineraloid *per se*) or if it is preferentially associated with specific (primary magmatic or secondary) minerals. MOMA LDMS can also help constrain the host mineral phase as the MOMA UV laser ablates both organic and inorganic fragments to be analyzed by the MS. Further constraints will come from MicrOmega and RLS. Both techniques are particularly diagnostic not only for inorganic minerals but can also detect some organic compounds. Therefore, the synergy of MOMA with these instruments is considerable in view of the overall goal of the mission (Vago *et al.*, 2017).

Finally, one of the most significant improvements of the ExoMars rover mission compared with previous missions is its capability to acquire samples from the subsurface down to a depth of about 2 m. This capability enables the mission to acquire and analyze samples that have been potentially shielded from solar and galactic radiation (mostly high-energy gamma radiation) over geologic timescales (Pavlov *et al.*, 2012) and that may contain highly pristine martian organic material, perhaps at much higher abundance than at the top surface.

## 9. Summary

The access to samples from up 2 m below the martian surface and the wide range of analytical capabilities will enable MOMA to play a major role in constraining potential sources of organic material in martian sediments and in constraining processes (be they of abiotic or biogenic nature) that might have altered this material. This new insight will inform the next step of Mars exploration: Mars Sample Return.

## Abbreviations Used

ADRON: Active Detector for gamma Rays and Neutrons
AGC: Automatic Gain Control (for laser output control)
ALD: Analytical Laboratory Drawer (containing the analytical instruments (MicrOmega, RLS, MOMA) and UCZ)
CDH: Command and Data Handling board (part of MEB)
CEM: Channel Electron Multiplier (part of MS detector)
CLUPI: CLose-Up Imager
COSAC: COmetary SAmpling and Composition (GCMS instrument onboard Philae lander that is part of the Rosetta mission to comet 67P)
CTL: Control board (part of SEB)
DMF: *N,N*-dimethylformamide (solvent for MTBSTFA and GC calibrant)
DMF-DMA: *N,N*-dimethylformamide-dimethylacetal (MOMA derivatization agent)
DSC: Differential Scanning Calorimetry
DTA: Differential Thermal Analysis
EDL: Entry Descent Landing
ee: enantiomeric excess (of chiral organic molecules)
EGA: Evolved Gas Analysis
EI: Electron Ionization Source (part of MS)
ETU: Engineering Test Unit (flight-like test unit for LDMS)
FAS: Flight Analog System (setup at MPS, replicating some aspects of GCMS)
FB: Filament Bias board (part of SEB)
FM: Flight Model
FS: Flight Spare
FWHM: Full-Width Half-Maximum
GC: Gas Chromatograph or Gas Chromatography
GCMS: Gas Chromatography–Mass Spectrometry
GSFC: Goddard Space Flight Center, Greenbelt (NASA center developing MS and electronics)
GT: Gas Tank (of GC system)
ISEM: Infrared Spectrometer for ExoMars
ITMS: Ion Trap Mass Spectrometry
LADEE: Lunar Atmosphere and Dust Environment Explorer (orbiter, September 2013 to April 2014)
LATMOS: Laboratoire ATmosphères, Milieux, Observations Spatiales, Guyancourt (developing GCMS and electronics)
LD: Laser Desorption
LDI: Laser Desorption and Ionization
LDMS: Laser Desorption Mass Spectrometry
LH: Laser Head
LISA: Laboratoire Interuniversitaire des Systèmes Atmosphériques, Créteil (developing GCMS and electronics)
LIT: Linear Ion Trap (part of MS)
LPU: Laser Power Unit (part of MEB)
LZH: Laser-Zentrum Hannover (developing UV laser for LDMS, MPS subcontractor)
MAIF: Motor and Analog Interface (part of MEB)
Ma_MISS: Mars Multispectral Imager for Subsurface Studies
MAVEN: Mars Atmosphere and Volatile EvolutioN (orbiter, since 2014)
MEB: Main Electronic Box
MER: Mars Exploration Rovers (since 2004)
MEX: Mars EXpress (orbiter since 2004)
MicrOmega: “Micro version” of OMEGA (ALD instrument)
MOMA: Mars Organic Molecule Analyzer (ALD instrument)
MPS: Max Planck Institute for Solar System Research, Göttingen (developing MOMA subsystems (oven, TP, laser) and electronics)
MS: Mass Spectrometer or Mass Spectrometry
MS/MS: tandem MS (MS operational mode)
MSL: Mars Science Laboratory (Curiosity Rover, since 2012)
MTBSTFA: *N,N*-methyl-tert.-butyl-dimethylsilyltrifluoroacetamide (MOMA derivatization agent)
OMEGA: Observatoire pour la Minéralogie, l'Eau, les Glaces et l'Activité (VIS/NIR mineralogical mapping spectrometer onboard MEX)
PAH: Polyaromatic Hydrocarbon
PanCam: Panoramic Camera System
PCV: Pressure Control Valve (controlling GC gas flow)
PFTBA: perfluorotributylamine
PH: Power and High-Voltage (part of SEB)
PS: Power Supply
QM: Qualification Model
QMS: Quadrupole Mass Spectrometer
QSM: Qualification Simulator Model
RF: Radio Frequency (power supply for LIT control)
RLS: Raman Laser Spectrometer (ALD instrument)
RSM: Reference Surface Mission (preliminary rover mission plan, feasibility study in terms of time, power, and data resources)
SAM: Sample Analysis at Mars (organic molecule and volatile analyzer onboard MSL)
SEB: Secondary Electronic Box
SNR: Signal-to-Noise Ratio
SPDS: Sample Processing and Distribution System (composed of sample inlet, sample path, and oven carousel, not part of MOMA)
SPRL: Space Physics Research Laboratory
SWIFT: Stored Waveform Inverse Fourier Transform (MS operational mode)
TAS-I: Thales Alenia Space in Torino, Italy
TCD: Thermal Conductivity Detector (GC detector)
TEGA: Thermal and Evolved Gas Analyzer (onboard the Phoenix Mars lander, May to October 2008)
TMAH: tetramethylammonium hydroxide (MOMA derivatization agent)
TS: Tapping Station (sealing the oven for GCMS)
UCZ: Ultra-Clean Zone (part of SPDS that is in direct contact with martian samples)
UV: ultraviolet
WISDOM: Water Ice Subsurface Deposit Observation on Mars (radar for shallow subsurface)
WRP: Wide-Range Pump
